# The occurrence of *Listeria monocytogenes* is associated with built environment microbiota in three tree fruit processing facilities

**DOI:** 10.1186/s40168-019-0726-2

**Published:** 2019-08-21

**Authors:** Xiaoqing Tan, Taejung Chung, Yi Chen, Dumitru Macarisin, Luke LaBorde, Jasna Kovac

**Affiliations:** 1 Department of Food Science, The Pennsylvania State University, University Park, PA 16802 USA; 2 Microbiome Center, Huck Institutes of the Life Sciences, The Pennsylvania State University, University Park, PA 16802 USA; 3 Center for Food Safety and Applied Nutrition, Food and Drug Administration, College Park, MD 20740 USA

**Keywords:** *Listeria monocytogenes*, Microbiota, Mycobiota, Fruit, Built food processing environment

## Abstract

**Background:**

Multistate foodborne disease outbreaks and recalls of apples and apple products contaminated with *Listeria monocytogenes* demonstrate the need for improved pathogen control in the apple supply chain. Apple processing facilities have been identified in the past as potential sources of persisting *L*. *monocytogenes* contamination. In this study, we sought to understand the composition of microbiota in built apple and other tree fruit processing environments and its association with the occurrence of the foodborne pathogen *L*. *monocytogenes*.

**Results:**

Analysis of 117 samples collected from three apple and other tree fruit packing facilities (F1, F2, and F3) showed that facility F2 had a significantly higher *L*. *monocytogenes* occurrence compared to F1 and F3 (*p* < 0.01). The microbiota in facility F2 was distinct compared to facilities F1 and F3 as supported by the mean Shannon index for bacterial and fungal alpha diversities that was significantly lower in F2, compared to F1 and F3 (*p* < 0.01). Microbiota in F2 was uniquely predominated by bacterial family Pseudomonadaceae and fungal family Dipodascaceae.

**Conclusions:**

The composition and diversity of microbiota and mycobiota present in the investigated built food processing environments may be indicative of persistent contamination with *L*. *monocytogenes*. These findings support the need for further investigation of the role of the microbial communities in the persistence of *L*. *monocytogenes* to support the optimization of *L*. *monocytogenes* control strategies in the apple supply chain.

**Electronic supplementary material:**

The online version of this article (10.1186/s40168-019-0726-2) contains supplementary material, which is available to authorized users.

## Background

Listeriosis is a foodborne infectious disease caused by *Listeria monocytogenes*. Listeriosis has 20% to 30% fatality rate in high-risk groups, such as elderly, pregnant woman, and immunocompromised individuals [1]. *L*. *monocytogenes* infections have been historically associated with outbreaks traced back to ready-to-eat meat products [2] and unpasteurized raw milk [3]. However, in recent years, an increased number of listeriosis outbreaks have been linked with contaminated fresh produce [4, 5]. In 2014, a multistate outbreak of listeriosis traced back to contaminated prepackaged caramel apples caused 34 hospitalizations and seven deaths [6]. In 2017, three more cases of listeriosis associated with prepackaged caramel apples were reported [7]. Several recalls of sliced apples contaminated with *L*. *monocytogenes* have raised broader concerns about the safety of apples, primarily when further processing provides suitable conditions for *L*. *monocytogenes* growth [8, 9]. Increased food safety scrutiny has led to enhanced monitoring of *L*. *monocytogenes* in tree fruit packing and processing facilities, and there is a need to investigate factors that may play a role in the establishment and persistence of *L*. *monocytogenes* in built environments in the apple supply continuum.

A number of studies have investigated the effects of commonly used sanitizers on the reduction of *L*. *monocytogenes* and on its ability to adapt to these antimicrobial treatments [10–13]. These studies provided valuable insight into the ability of sanitizers to inhibit the growth of *L*. *monocytogenes* in monocultures [10, 14]; however, they failed to adequately model the complex biotic environmental conditions found in post-harvest food processing built environments [15, 16]. It is becoming increasingly evident that microbiota found in food production and processing environments plays a role in pathogen survival and persistence and that microbiota needs to be taken in consideration when assessing the effectiveness of pathogen control strategies [16].

Interspecies interactions between *L*. *monocytogenes* and other microorganisms that make up the food processing environment microbiota have been shown to alter the ability of *L*. *monocytogenes* to survive and colonize facilities [17]. For example, *Pseudomonas* spp. commonly found in food processing environments [14, 15] has been found to have a positive effect on *L*. *monocytogenes* attachment on the stainless steel surfaces [18]. In contrast, *Staphylococcus sciuri* was shown to decrease *L*. *monocytogenes* biofilm formation on stainless steel surfaces [19]. Carpentier and Chassaing [20] demonstrated that among 29 bacteria isolated from the dairy and meat processing environment, four strains promoted *L*. *monocytogenes* growth in the resulting biofilm when co-cultured with *L*. *monocytogenes*, whereas 16 strains suppressed the growth of *L*. *monocytogenes*. Although the exact mechanisms underlying these biological interactions and phenotypic outcomes are yet to be elucidated, competition for nutrients and production of anti-listerial secondary metabolites have been proposed as important microbiota-shaping factors [19, 21]. One of the important steps toward gaining a better understanding of microbial interactions in microbial communities is the characterization of environmental microbiota in food production and processing facilities and investigation of associations and co-occurrence of *L*. *monocytogenes* and other members of the microbiota.

Amplicon sequencing has revolutionized the characterization of microbial communities, not only in human medicine and ecology but also in the food industry where information about microbiota dynamics can enhance our ability to answer applied questions related to food safety and quality. Microorganisms such as *P*. *psychrophila*, *Pseudomonas* sp., *Klebsiella* sp., *K*. *oxytoca*, and *Aeromonas hydrophila* have been identified through 16S rRNA sequencing as the dominant species in *L*. *monocytogenes*-positive drains located in dairy, meat, peanut butter, and spice processing plants, suggesting that they might facilitate *L*. *monocytogenes* biofilm formation through interspecies interactions [22]. Moreover, studies have been carried out in dairy and meat processing plants, where associations have been found between the indoor bacterial communities and the presence of *L*. *monocytogenes* [23, 24]. Food processing facilities that serve as an intermediate between the pre-harvest, raw ingredient, and retail distribution chain have been identified among the main potential sources of pathogen contamination [25–28]. It is therefore critical to establish a baseline understanding of microbial diversity in food processing environments and to investigate the associations between microbial community composition and occurrence of *L*. *monocytogenes* in these environments. This knowledge can be used to develop targeted microbiota manipulation strategies for the development of improved foodborne pathogen control strategies.

There is only limited data available on the composition of microbial communities in built produce processing environments [29]. To the best of our knowledge, no published studies have reported the relationship between apple and other tree fruit packing house microbiota composition and the presence of *L*. *monocytogenes* in these environments to date. We, therefore, utilized 16S rRNA V4 and internal transcribed spacer 2 (ITS2) amplicon sequencing coupled with *L*. *monocytogenes* enrichment to elucidate the associations between the composition, diversity, and predicted functional profiles of the built environment microbiota in three apple and other tree fruit packing facilities to provide new knowledge enabling improvement of food safety.

## Methods

### Study design

Occurrence of *Listeria monocytogenes* and characterization of microbiota within three apple and other tree fruit packing houses in the Northeast U.S. was monitored through the fruit harvesting and packing season. At each facility, nonfood-contact (zone 3) environmental samples were collected from the floor under a conveyor system with rolling brushes that transported fruit through successive washing, drying, and waxing processes, as outlined below. These specific locations were selected for sampling because preliminary data from season 2016/17 showed a higher occurrence of *L*. *monocytogenes* in these locations compared to other locations in the packing houses. Based on the preliminary data, we established that a sample size of 117 is needed to identify the prevalence of *L*. *monocytogenes* with a 7.25% precision and 95% confidence [30]. Therefore, a total of 117 samples, 39 samples from each of the three facilities, were collected twice a month over 13 sampling time points between November 2017 and April 2018. Information including the facility code, sampling date, and sampling location within the facility are provided in the Additional file 1: Table S1.

### Sample collection

**Fig. 1:**
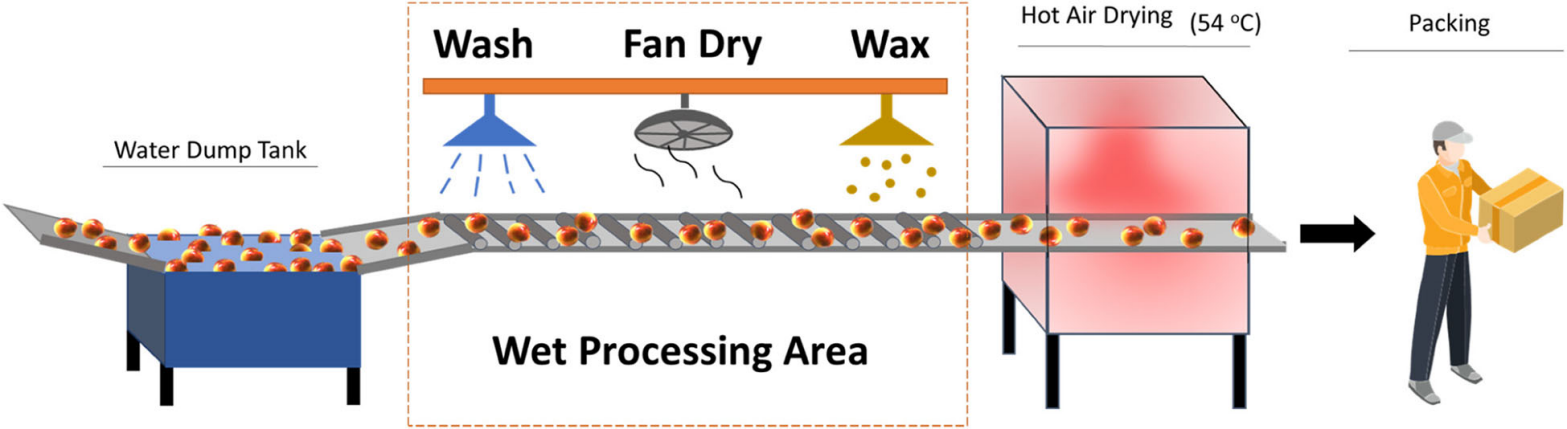
Apple processing workflow representative of monitored apple and other tree fruit packing facilities. Samples were collected in the wet processing area under the rotating brush conveyor belt that consisted of washing, drying, and waxing area

Non-food contact surface environmental areas (40 cm by 40 cm) were sampled by swabbing with pre-moistened sponges (3M) with a combination of ten horizontal and ten vertical strokes. Samples for *Listeria* spp. isolation and identification were collected with 3M hydrated sponges pre-moistened with 10 ml D/E neutralizing buffer (3M, cat. number HS10DE2G) to enhance the survival of *Listeria* through the neutralization of a broader range of sanitizers [31]. The samples for microbiota characterization were collected using 3M hydrated sponges with just 10 ml of neutralizing buffer (3M, cat. number HS2410NB2G). Two adjacent duplicate areas were sampled at each of the three areas (washing, drying, waxing) under the conveyor belt (Fig. 1). One of the duplicate samples was used for *L*. *monocytogenes* enrichment, isolation, and identification, and the other for microbiota and mycobiota characterization using 16S rRNA V4 and ITS2 Illumina sequencing. A total of 18 samples were collected per time point; six in each of the three facilities. All swab samples were stored in a cooler on the ice during transportation to the laboratory and were processed on the same day.

### *Listeria monocytogenes* enrichment and isolation

Detection of *Listeria* spp. was conducted following a modified Food and Drug Administration bacteriological analytical manual (FDA BAM) protocol for the detection and enumeration of *L*. *monocytogenes* [31]. Briefly, 90 ml of buffered *Listeria* enrichment broth (BLEB, Oxoid) were added to each test sample and control sample, followed by manual homogenization by hand-massaging the sponge for 15 s. *L*. *monocytogenes* strain F2365 [32] and *Listeria innocua* strain PS00298 were used as positive and negative controls in every sample processing batch, respectively, to compare their morphology with the morphology of putative *Listeria* spp. isolated on selective agars as well as with the resulting polymerase chain reaction (PCR) product separated using gel electrophoresis. A sterile sealed sampling sponge from the same production batch served as an additional negative control to ensure that the used batch of sampling sponges was not contaminated with *L*. *monocytogenes*. For the enrichment, 400 μl of BLEB supplement SR0149 (Oxoid) were added to each test sample and controls after 4 h of pre-enrichment in BLEB at 30 °C. After adding the supplement, samples were further incubated for 44 ± 2 h at 30 °C. Upon completed incubation, a loopful of each enrichment was streaked onto Agar *Listeria* Ottavani & Agosti (ALOA, BioRad Laboratories Inc.) and RAPID’ *L*. *mono* (BioRad Laboratories Inc.) selective differential agars, and incubated for 48 h at 37 °C. After incubation, presumptive positive *L*. *monocytogenes* colonies appeared blue/green with an opaque white halo on the ALOA agar, whereas presumptive positive *L*. *monocytogenes* colonies appeared blue with no background change on RAPID’ *L*. *mono* agar. Other *Listeria* spp. may also grow on these selective differential agars; however, only one presumptive *L*. *monocytogenes* colony was collected from each, ALOA and RAPID *L*’ *mono* agars, and streaked for isolation on trypticase soy agar with yeast extract (TSAYE) in this study. Streaked plates were incubated for 24 h at 37 °C and isolated colonies were used for PCR-based *L*. *monocytogenes* confirmation.

### *Listeria monocytogenes* identification and cryopreservation

One isolated colony per TSAYE plate was selected, inoculated in the trypticase soy broth with yeast extract (TSBYE), and incubated at 30 °C for 24 h. One milliliter of each overnight culture was centrifuged at 15,000×*g* for 15 min, washed with DNase free water, and centrifuged again at 15,000×*g* for 15 min. The cell pellets were resuspended in 50 μl of DNase free water and heated for 10 min at 95 °C to lyse the cells. Lysates were then centrifuged at 15,000×*g* for 10 min to remove the cell debris. Supernatants containing deoxyribonucleic acid (DNA) template were stored at − 20 °C until further use. Two microliters of DNA template were used in a PCR reaction with primers targeting genes *iap* (specific for *Listeria* spp.) and *lmo2234* (specific for *L*. *monocytogenes*) to confirm *the L*. *monocytogenes* species [32]. *L*. *monocytogenes* strain F2365 and *Listeria innocua* strain PS00298 served as positive controls and the nuclease-free water was used as a negative control. The following thermal cycling conditions were used: initial denaturation at 95 °C for 15 min, 15 cycles of denaturation at 94 °C for 1 min, annealing at 55 °C to 51 °C for 1 min with a touchdown of 3 cycles per temperature, extension at 72 °C for 1 min. The following 15 cycles started with denaturation at 94 °C for 1 min, annealing at 50 °C for 1 min, extension at 72 °C for 1 min, and the final extension at 72 °C for 8 min [32]. Successful PCR amplification was confirmed by gel electrophoresis using 2% agarose gel (Invitrogen). A band between 1450 and 1600 bp (for *iap*) and a band at 420 bp (specific for *lmo2234*) were expected in samples positive for *Listeria* spp. or *L*. *monocytogenes*, respectively. Isolates that were confirmed as *Listeria* spp. or *L*. *monocytogenes* were re-streaked on TSAYE and grown overnight in TSBYE. Overnight cultures were supplemented with 20% glycerol and stored in − 80 °C freezer.

### Quantification of *Listeria* using the most probable number assay

Most probable number (MPN) assay for *L*. *monocytogenes* was carried out on 18 samples collected in two samplings conducted in March 2018 to determine the level of *L*. *monocytogenes* contamination, using the enrichment media described above. Briefly, 90 ml of BLEB were added to a bag containing a sponge sample, and manually homogenized. One milliliter of the homogenate was added directly to 9 ml of BLEB, in triplicate, and enriched. Another 1 ml was serially tenfold diluted four times in 9 ml of PBS, in triplicates. One milliliter of each of these four serial dilutions were transferred into test tubes containing 9 ml of BLEB and enriched. This resulted in five MPN dilutions ranging from 10^−2^ (1/100 of homogenate) to 10^−6^ of the original sample. The remaining homogenate was enriched as well. Forty microliters of BLEB supplement were added to each tube after 4 h of incubation at 30 °C. Supplemented samples were further incubated for 44 ± 2 h at the same temperature. After 48-h incubation, a loopful of each dilution enrichment was streaked onto ALOA and RAPID’ *L*. *mono* agar plates and incubated at 37 °C for 24 to 48 h, until obtaining visible colonies. The MPN of *L*. *monocytogenes* per sponge sample was determined using an MPN calculator provided in the Excel file available for download in “BAM: Detection and Enumeration of *Listeria monocytogenes*” [33].

### Total DNA extraction for microbiota and mycobiota analysis

Each environmental sponge sample collected for microbiota characterization was homogenized with 50 ml of phosphate buffer containing 0.9% NaCl in a stomacher, for 7 min at 230 rpm. Fifty milliliters of the homogenate were transferred to a sterile 50 ml conical tube and centrifuged at 11,000×*g* and 4 °C for 20 min (Beckman Coulter, Avanti J-26 XPI) [34]. After centrifugation, supernatants were discarded and pellets were stored at − 80 °C until DNA extraction. DNA was extracted from approximately 0.25 g of each sample using DNeasy PowerSoil DNA extraction kit (QIAGEN) following manufacturer’s protocol. Approximately 0.25 g of a sterile sponge was also sampled and used as a negative control to confirm the absence of microbial DNA contaminants on the sterile sponge. DNA extracted from the sponge was processed following the same protocol as described below for other samples. The concentration of DNA in each test sample and in the control sample was determined both spectrophotometrically using NanoDrop One (Thermo Scientific) and fluorometrically using Qubit 3 (Invitrogen) and Qubit dsDNA High Sensitivity Assay Kit. DNA samples were stored at − 80 °C until further use.

### 16S rRNA V4 and ITS2 sequence amplification

Bacterial and fungal community composition was determined by targeted metagenomic sequencing of the PCR-amplified V4 domain of the 16S rRNA gene and the internal transcribed spacer 2 (ITS2) sequences, respectively. Briefly, V4 region of the 16S rRNA gene sequence was amplified using KAPA HiFi HotStart ReadyMix (Kapa Biosystems), a forward primer 505F-v2 (TCG TCG GCA GCG TCA GAT GTG TAT AAG AGA CAG GTG YCA GCM GCC GCG GTA A), and a reverse primer 806R-v2 (GTC TCG TGG GCT CGG AGA TGT GTA TAA GAG ACA GGG ACT ACN VGG GTW TCT AAT) [35]. PCR thermal cycling for amplification of the 16S rRNA gene V4 region was conducted as follows: initial denaturation at 95 °C for 3 min, 29 cycles of denaturation at 98 °C for 30 s, annealing at 55 °C for 30 s, extension at 72 °C for 30 s, and the final extension at 72 °C for 5 min, and final hold at 4 °C. Fungal internal transcribed spacer (ITS) loci were amplified using a forward primer ITS4F (TCG TCG GCA GCG TCA GAT GTG TAT AAG AGA CAG GAA CGC AGC RAA IIG YGA) and a reverse primer ITS9R (GTC TCG TGG GCT CGG AGA TGT GTA TAA GAG ACA GTC CTC CGC TTA TTG ATA TGC) [36]. PCR conditions for ITS amplification were as follows: initial denaturation at 98 °C for 5 min, 30 cycles of denaturation at 95 °C for 45 s, annealing at 50 °C for 60 s, extension at 72 °C for 60 s, and the final extension at 72 °C for 5 min, and the final hold at 4 °C. PCR amplicons were visualized by running gel electrophoresis using a 2% agarose gel to confirm successful amplification of target sequences.

### Amplicon library preparation and amplicon sequencing

Amplicon libraries were prepared based on Illumina’s 16S Metagenomic Sequencing Library Preparation protocol [37]. 16S rRNA V4 and ITS2 PCR amplicons were barcoded with unique combinations of i7 and i5 index adaptors (Integrated DNA Technologies) in a second-step PCR using following thermal cycling conditions: initial denaturation at 95 °C for 3 min, 8 cycles of denaturation at 95 °C for 30 s, annealing at 55 °C for 30 s, extension at 72 °C for 30 s, and final extension at 72 °C for 5 min; final hold was at 4 °C. Barcoded PCR amplicon libraries were purified twice with AmPure XP beads (Beckman Coulter) and then normalized using Mag-Bind EquiPure Library Normalization Kit (Omega Bio-tek) following manufacturer’s protocol. Concentrations of a subset of normalized libraries were verified using a high sensitivity double-stranded DNA kit with Qubit 3. Libraries were then pooled in equal volumes of 4 μl. The distribution of pooled library fragment sizes was verified using Bioanalyzer and its concentration using qPCR. Estimated amplicon lengths of 359 bp and 425 bp were used in the calculation of molar concentration and normalization. The library pool was denatured by diluting 5 μl of 4 nM library pool with 5 μl freshly prepared 0.2 N NaOH. The denatured library pool was diluted with a pre-chilled HT1 buffer to 7.5 pM with 10% PhiX internal control library. A total of 600 μl of denatured library pool spiked with PhiX was loaded onto the Illumina Miseq flow cell. Five hundred cycle V2 Illumina sequencing kit was used for 250 bp paired-end sequencing in two Illumina MiSeq sequencing runs. Sequencing reads were deposited in NCBI SRA under BioProject PRJNA527988.

### Sequence analyses and OTU normalization

Sequences were analyzed with Mothur v1.39.5 following protocols described in Schloss et al. [38, 39] with default settings unless otherwise noted. The workflow of analyses is described in the Additional file 1: List L1. Paired end sequence reads were assembled into contigs. Contigs shorter or longer than 292 bp for 16S rRNA V4 bacterial sequences and longer than 350 bp for ITS2 fungal sequences were discarded. The remaining reads were aligned against SILVA database [40] for 16S rRNA V4 region of bacterial sequences, and UNITE database [41] for ITS2 fungal sequences. Chimera were detected and discarded using UCHIME algorithm. Operational taxonomic units (OTUs) were calculated using opticlust with 97% similarity threshold. All 16S rRNA sequences were rarefied to 4501 and ITS sequences were rarefied to 5232 randomly sampled OTUs. Rarefied sequence cutoffs were chosen based on the lowest reads obtained for microbiota and mycobiota samples, in order to include all samples in downstream comparative analyses.

### Diversity of microbiota and mycobiota

Alpha and beta diversity indices were calculated based on rarefied OTUs. For alpha diversity, Shannon and Inverse Simpson indices were calculated for both bacterial and fungal communities. For beta diversity, the weighted UniFrac distance between either bacterial or fungal communities was calculated in R using package Phyloseq [42]. Principal coordinates analysis (PCoA) was used to visualize the beta diversity of microbiota with reduced dimensionality. Principal coordinates were plotted using R package ggplot2 [43]. Chi-square and Fisher’s exact tests with Bonferroni correction were used to test the significance of differences in *L*. *monocytogenes* occurrence among tree frui packing facilities. Pairwise permutational multivariate analysis of variance (PERMANOVA) test was carried out using package PairwiseAdonis [44] and Bray-Curtis dissimilarity matrices were used to test the significance of differences in microbiota and mycobiota composition. Pairwise comparisons were carried out by sampling month, processing section, and facility.

### The co-occurrence of microbial and fungal taxa

Network analyses were carried out to identify significantly co-occurring bacterial families among samples collected from three apple and other tree fruit packing facilities. Networks were constructed using Cytoscape v3.7.1 [45] with CoNet version 1.1.1 beta plug-in [46]. Rarefied 16S rRNA OTU table was condensed to family level taxa and used as an input matrix. Network analysis was conducted based on microbial family occurrence for each individual facility as well as for all three facilities combined. Co-occurrence of specific families was determined using Pearson and Spearman correlations, mutual information, and Bray-Curtis, and Kullback-Leibler dissimilarity indices. Row-shuffle randomization and bootstrap method were used to minimize composition-induced false correlations. The resulting *p* values were merged using Brown’s method and corrected by Benjamini-Hochberg multiple comparison corrections. Interactions were visualized using yfiles layout algorithm plug-in application [47].

### Prediction of microbiota’s functional profiles

Raw 16S rRNA sequence reads were re-analyzed using Mothur pipeline outlined above, only this time in conjunction with Greengenes 16S rRNA database version13.8 [48] since Phylogenetic Investigation of Communities by Reconstruction of Unobserved States (PICRUSt) [49] required the use of this database. The biom file resulting from Mothur analysis, and containing OTU and taxonomy information was used as an input file for PICRUSt analysis using online Galaxy-based version provided by Hutlab [50]. Briefly, OTU table was first corrected for 16S rRNA copy number by dividing OTU counts by the number of 16S rRNA marker gene copy to obtain an abundance estimation for each OTU. Each Kyoto Encyclopedia of Gene and Genomes (KEGG) [51] ortholog (KO) was then multiplied by the normalized OTU abundance for metagenome prediction. KO tier 3 was assigned for functional prediction and tier 1 assignments were used for the categorization of functional profiles for each facility. The output data table contained summed predicted KO functional gene abundance per metagenome sample.

## Results

### Samples collected from facility F2 had higher occurrence and level of *L*. *monocytogenes* compared to those collected from F1 and F3

**Fig. 2:**
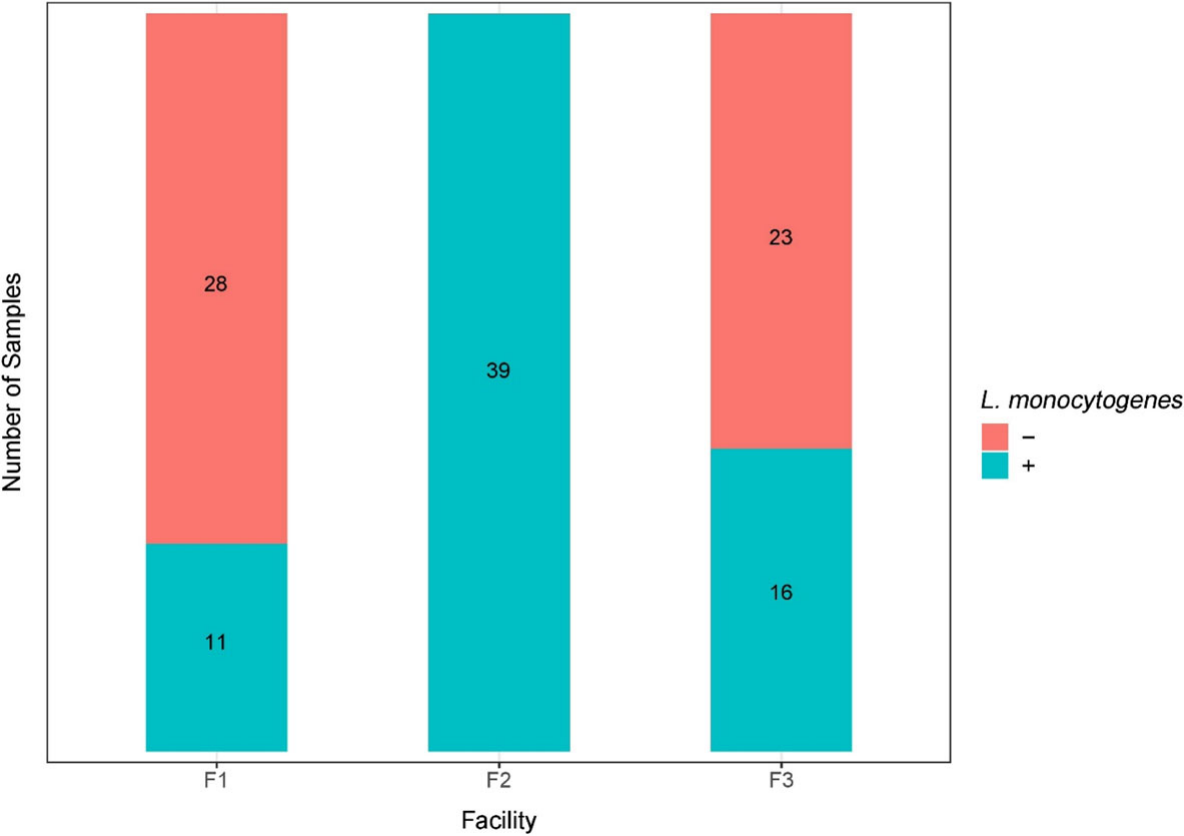
Summary of the presence (+) and absence (−) of *Listeria monocytogenes* in environmental samples collected from three tree fruit packing facilities F1, F2, and F3 over the sampling period (November 2017–April 2018), as determined using an enrichment protocol

A total of 117 environmental samples were tested for the presence of *L*. *monocytogenes* using the FDA BAM enrichment protocol. Out of 39 samples collected in each facility, *L*. *monocytogenes* culture was isolated and confirmed in 11, 39, and 16 samples from facilities. F1, F2, and F3, respectively (Fig. 2). All samples in facility F2 were positive for *L*. *monocytogenes* using the culturing method, while 11 (28%) and 16 (41%) samples were positive in facilities F1 and F3, respectively. The difference in occurrence of *L*. *monocytogenes* among facilities was statistically significant (*p* < 0.001). However, there was no significant difference in *L*. *monocytogenes* occurrence when comparing washing, fan-drying, and waxing sections (*p* = 0.112). Results of statistical analyses are provided in Additional file 1: Tables S2 and S3.

Quantification of *L*. *monocytogenes* in a subset of samples collected on 2 days in March 2018 indicated high-level contamination of F2 at both sampling times. Samples collected underneath the conveyor belt in washing, fan-drying, and waxing sections of the facility F2 contained an average of 3.51, 4.18, and 4.87 log_10_MPN/sponge, while an average of 1.50, 0.82, and 2.00 log_10_MPN/sponge were found in respective areas in facility F3. No *L*. *monocytogenes* was detected in samples collected from facility F1 in the two sampling time points (Table 1). Samples collected from three facilities had significantly different levels of *L*. *monocytogenes* (*p* < 0.001), with samples collected from F2 having higher-level of contamination compared to those collected from F1 and F3.

**Table 1:** MPN-based quantification of *L*. *monocytogenes* in samples collected from three tree fruit packing facilities at two time points in March 2018

Log MPN/sponge sample	Facility F1^c^			Facility F2			Facility F3^c^		
	Wash	Dry	Wax	Wash	Dry	Wax	Wash	Dry	Wax
Trial 1^a^	< LD	< LD	< LD	3.87	5.02	4.38	LD < 1.55^d^	< LD	LD < 1.55^d^
Trial 2^b^	< LD	< LD	< LD	3.15	3.32	5.35	1.55	1.55	2.63
Average	< LD	< LD	< LD	3.51	4.17	4.87	< 1.55	0.82	< 2.63

^a^Four dilutions (10^−2^, 10^−3^, 10^−4^, 10^−5^) were used in the MPN calculation. Each dilution was tested in triplicates. Sample homogenate (a sponge in 90 ml of BLEB) was also enriched

^b^Five dilutions (10^−2^, 10^−3^, 10^−4^, 10^−5^, 10^−6^) were used in MPN calculation. Each dilution was tested in triplicates. Sample homogenate (a sponge in 90 ml of BLEB) was also enriched

^c^< LD indicates that *L*. *monocytogenes* was below the limit of detection, which was at least one viable cell per sample, grown to at least 10^5^ cells/ml by the time the enrichment was completed

^d^The level of *L*. *monocytogenes* was between the limit of detection and 1.55 log_10_ MPN/sponge sample. *L*. *monocytogenes* was detected in enriched sponge homogenate; however, none of the MPN dilution tubes were positive

### 16S rRNA and ITS2 were rarefied to the lowest OTU count prior to downstream analyses

In the first sequencing run, a total of 8,298,240 16S rRNA V4 reads and 8,688,706 ITS2 reads were produced. Samples that were sequenced with fewer than 5000 reads were re-sequenced in a second sequencing run. In the second sequencing run, 7,458,554 16S rRNA reads and 3,485,400 ITS2 reads were produced. For16S rRNA sequences, a minimum, median, and maximum number of total OTUs identified was 4501, 28,056, and 168,219, respectively, and for ITS2 sequences, a minimum, median, and maximum numbers of total OTUs identified were 5323, 17,882, and 350,415.

**Fig. 3:**
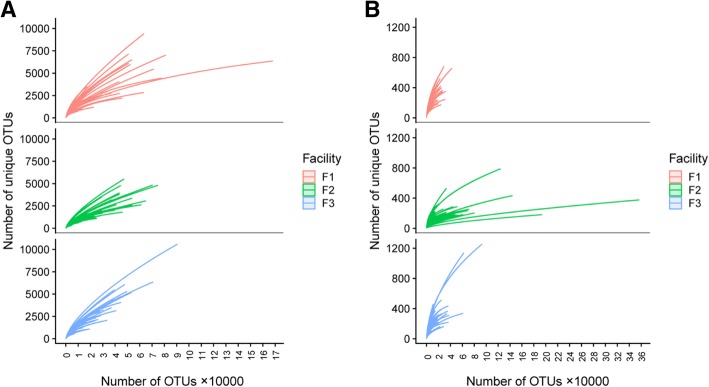
Rarefaction curves for 16S rRNA V4 (**a**) and ITS2 (**b**) sequences. Individual samples were plotted using the number of OTUs that represent the sequence sample size on *x* axis and the number of unique OTUs that indicate the species richness on *y* axis. Each curve is color-coded based on the facility in which the sample was collected

Rarefaction curves were plotted using the number of OTUs which represented the sequence sample size, against the number of unique OTUs which represented species richness (Fig. 3). All three facilities generally had a similar sequence sample size; however, richness in samples from facility F2 16S rRNA reached saturation at fewer reads compared to facilities F1 and F3, indicating lower microbial diversity in F2. For ITS2 OTUs, samples from facility F2 had a substantially higher numbers of assembled OTUs compared to samples from F1 and F3. The rarefaction curves for samples from facilities F1 and F3 did not reach saturation, indicating that considerable microbial diversity of low-abundant taxa has not been discovered at a given sequencing depth. Based on the rarefaction curves and our interest in high-abundant taxa that were hypothesized to play a major role in the microbial ecology of monitored environments, we rarefied samples to the lowest number of total OTUs found among sequenced samples (i.e., *N* = 4501 for 16S rRNA V4 sequences and *N* = 5323 for ITS2 sequences).

### Alpha diversity of bacterial communities was significantly different in samples collected from facility F2 compared to those collected from facilities F1 and F3

**Fig. 4:**
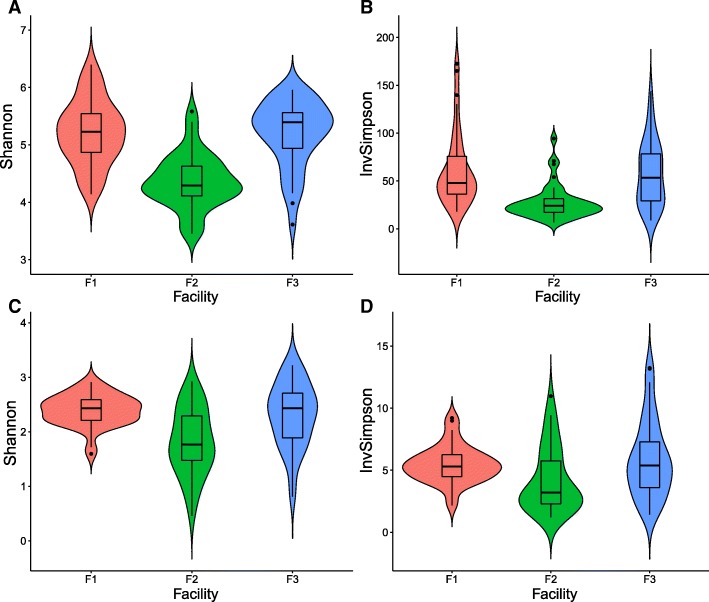
Microbial alpha diversity distributions determined based on rarefied 16S rRNA V4 and ITS2 assembled contigs for samples collected from three packing facilities, F1, F2 and F3. Alpha diversity was measured using Shannon index (**a**) and Inverse Simpson index (**b**). Fungal alpha diversity was determined based on rarefied ITS2 assembled contigs, as measured using Shannon index (**c**) and Inverse Simpson index (**d**)

Microbial and fungal composition of environmental samples collected from three facilities were characterized using rarefied 16S rRNA V4 and ITS2 amplicon sequencing. Alpha diversity indices were visualized using violin plots (Fig. 4) showing microbiota and mycobiota diversity within each individual facility. Significant differences in bacterial alpha diversity were observed among microbiota of samples collected in facilities F1, F3, and F2. Pairwise *t* test comparison showed no significant difference in microbial alpha diversity determined based on Inverse Simpson index (Fig. 4a) between facilities F1 and F3 (*p* = 1.00), while significant difference was observed between diversity of microbiota from facilities F1 and F2 (*p* = 1.7 × 10^−5^), as well as from facilities F3 and F2 (*p* = 1.5 × 10^−4^). Shannon index showed similar trends when comparing the alpha diversity of samples from F1 with samples from F3 (*p* = 1.00). Alpha diversities of samples from F1 (*p* = 6.0 × 10^−10^) and F3 (*p* = 1.5 × 10^−9^) were significantly different from those from facility F2, according to the Shannon index (Fig. 4b).

Alpha diversity indices for fungal communities were also compared among samples collected in three facilities. Significant differences were identified between samples from facilities F1 and F2 (*p* = 7.1 × 10^−6^) as well as samples from F2 and F3 (*p* = 1.6 × 10^−4^) as indicated by the Shannon index. No significant difference was observed between the alpha diversity of samples from F1 and F3 (*p* = 1.0, with Bonferroni correction, Fig. 4d). Inverse Simpson index, on the other hand, showed no significant difference between samples from F1 and F2 (*p* = 0.082) nor F1 and F3 (*p* = 1.00). A significant difference, however, was observed between F2 and F3 (*p* = 0.011) (Fig. 4c). Overall, the microbial diversity in facility F2 was substantially lower compared to that in facilities F1 and F3.

### Environmental microbiota in facility F2 significantly differed from environmental microbiota in facilities F1 and F3

**Fig. 5:**
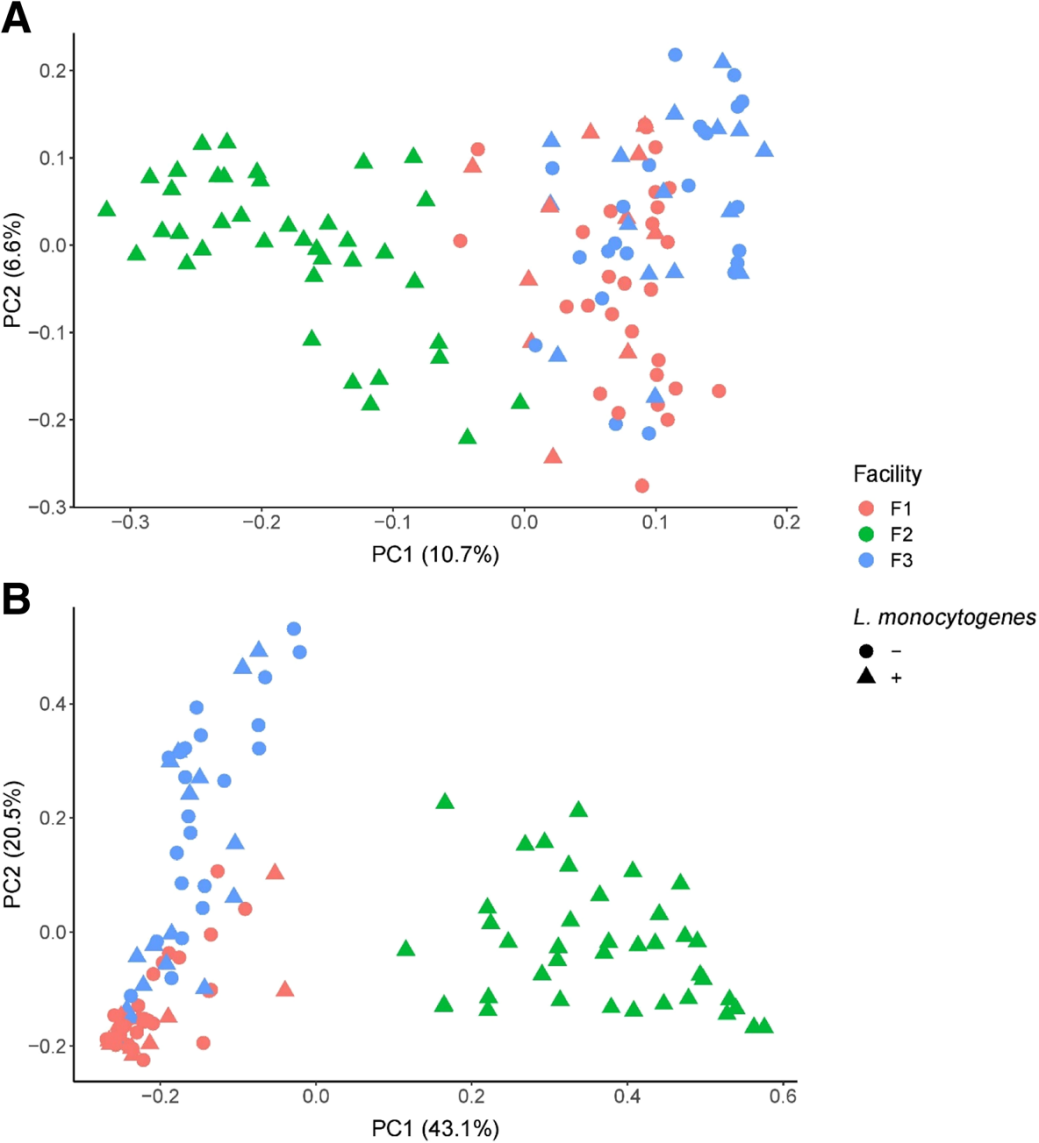
Clustering of bacterial (**a**) and fungal (**b**) communities based on the UniFrac distances calculated using 16S rRNA V4 gene sequences for bacteria and ITS2 sequences for fungi. The colors red, green, and blue indicate samples from facilities F1, F2 and F3, respectively. The circles and triangles indicate samples positive (+) or negative (−) for *L*. *monocytogenes* culture, respectively

The beta diversity of bacterial communities among facilities was shown in the PCoA plot (Fig. 5). Clustering was analyzed using weighted UniFrac metric which incorporates phylogenetic relatedness when calculating distance among 16S rRNA or ITS2 gene sequences. Further, 10.7% and 6.6% variances in bacterial composition were represented by the PC1 and PC2, respectively. Moreover, 43.1% and 20.5% variability in the beta diversity were explained by the PC1 and PC2 for mycobiota. Microbiota of samples collected from facility F2 formed a distinct cluster while microbiota of samples collected from facilities F1 and F3 appeared to be more related to each other than to microbiota from F2 (Fig. 5a). Consistently with distinct F2 microbiota clustering, mycobiota from this facility also appeared to be different compared to that discovered in facilities F1 and F3 (Fig. 5b). However, unlike microbiota, the mycobiota found in samples from facilities F1 and F3 appeared to be less similar (Fig. 5b). In order to further explore the microbial composition that contributed to the distinct clustering of microbiota in the PCoA plot, we investigated the taxonomic composition of collected samples in three individual facilities using PERMANOVA statistical analyses.

### Pairwise PERMANOVA analysis indicated that significant differences in microbiota composition exist among facilities

Beta diversity plot indicated distinct clustering of microbiota among facilities. To statistically evaluate these potential differences, we compared microbiota compositions by the month of sample collection, sampling section, and the individual facility using pairwise PERMANOVA. Our results indicate that the composition of the microbiota did not vary significantly by month (*p* > 0.05, Additional file 1: Table S4). However, we found that different facilities and sections had a significant effect on the composition of microbiota (*p* < 0.003; Table 2). Mycobiota composition was also not significantly different in different months (Additional file 1: Table S5), while it was significantly different in different facilities (Table 2). Moreover, when comparing samples from different processing sections, mycobiota in washing and fan-drying, as well as in fan-drying and waxing sections was not significantly different (*p* = 0.057 and 1.00, respectively). However, mycobiota differed significantly between washing and waxing sections (*p* = 0.012; Additional file 1: Table S3).

**Table 2:** Comparison of microbiota and mycobiota composition among different facilities

Facility pair	DF^a^	Sum of squares	*F* model	*R* ^2b^	*P* value	*P* adjusted	Sig^c^
Microbiota							
F1 vs F2	1	2.788	7.664	0.092	0.001	0.003	A
F1 vs F3	1	1.525	3.904	0.049	0.001	0.003	B
F2 vs F3	1	3.259	8.983	0.106	0.001	0.003	C
Mycobiota							
F1 vs F2	1	10.111	82.072	0.519	0.001	0.003	A
F1 vs F3	1	3.247	23.382	0.235	0.001	0.003	B
F2 vs F3	1	8.281	65.764	0.464	0.001	0.003	C

^a^*DF* degree of freedom

^b^*R*^2^ R square

^c^Sig, letter indicates significant difference between facility pairs

### Communities of environmental samples from facility F2 are predominated by Pseudomonadaceae and Dipodascaceae

**Fig. 6:**
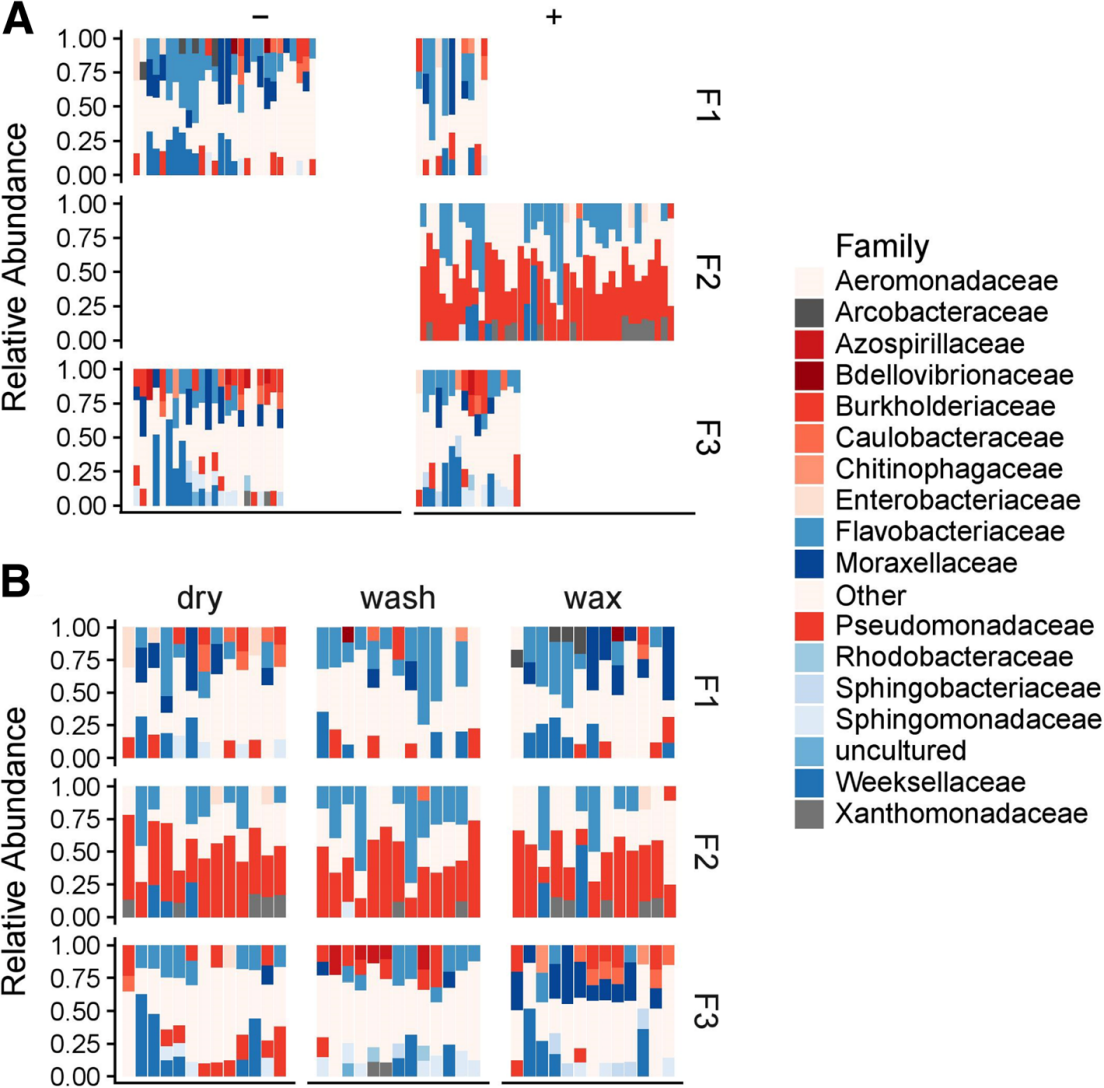
Relative abundances of bacterial families in samples collected in three tree fruit packing facilities, shown by the presence (+) or absence (−) of *L*. *monocytogenes* culture in facilities F1, F2, and F3 (**a**) and in three sections (washing, fan drying, and waxing) within facilities (**b**). Families with relative abundance of less than 10% are shown under the category “Other.” Each bar represents an individual sample

To explain the differential clustering of microbiota from samples collected in facility F2, we examined the taxonomic composition of the samples from different facilities (Fig. 6). Facilities F1, F2, and F3 contained 12, 8, and 14 unique bacterial families that were present at 10% or higher relative abundance (RA), respectively (Fig. 6). Families present in less than 10% relative abundance were grouped in a category “Other.” The top three most abundant families found in facility F1 were Flavobacteriaceae (19.68%), Moraxellaceae (11.93%), and Weeksellaceae (10.27%). High abundance of Pseudomonadaceae was found in facility F2 (41.97%), where Flavobacteriaceae (18.27%) and Xanthomonadaceae (7.69%) were also relatively highly abundant. Facility F3 had highly abundant Weeksellaceae (13.22%), Flavobacteriaceae (11.45%) and both Burkholderiaceae and Moraxellaceae were present at 8.67%. Pseudomonadaceae was identified as a predominant family in samples from facility F2 (41.97% RA), which also had the highest occurrence of *L*. *monocytogenes*. Samples from facility F2 had significantly higher RA of Pseudomonadaceae compared to facilities F1 and F3 which contained 7.76% and 6.48% Pseudomonadaceae respectively (*p* < 0.0001). There was no significant difference in Pseudomonadaceae abundance between facilities F1 and F3 (*p* = 0.844). Another bacterial family that was frequently identified in high relative abundance in facility F2 was Flavobacteriaceae; however, it was frequently present in high relative abundance also in samples from facilities F1 and F3.

**Fig. 7:**
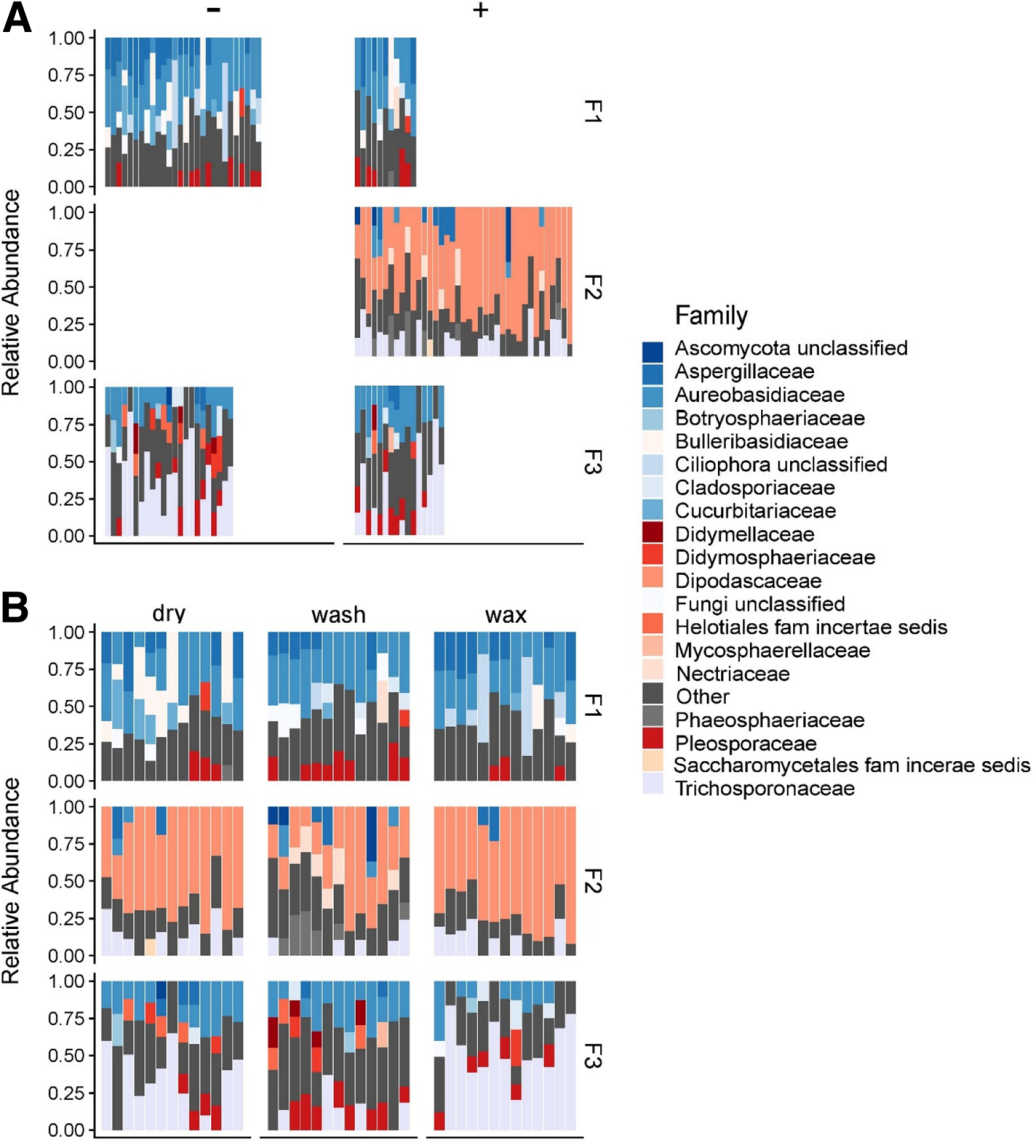
Relative abundances of fungal families in samples collected in three tree fruit packing facilities, shown by the presence (+) or absence (−) of *L*. *monocytogenes* culture in facilities F1, F2, and F3 (**a**) and in three sections (washing, fan drying, and waxing) within facilities (**b**). Families with relative abundance less than 10 % are shown under the category “Other.” Each bar represents an individual sample

We further explored the difference in fungal communities among three monitored facilities by comparing mycobiota taxonomy at a family level (Fig. 7). The top three abundant families found in facility F1 were Aureobasidiaceae (30.04%), Aspergillaceae (12.69%), and Bulleribasidiaceae (9.50%). Facility F2 had three highly abundant families, including Dipodascaceae (56.08%), Trichosporonaceae (10.88%), and Aspergillaceae (4.52%), and the most abundant three fungal families found in facility F3 included Trichosporonaceae (30.63%), Aureobasidiaceae (18.2%), and Pleosporaceae (9.9%). A higher abundance of Dipodascaceae was observed in facility F2, whereas it was present at very low RA in both facilities F1 (0.93%) and F3 (0.48%) (*p* < 0.01). In contrast, Aureobasidiaceae was present at a relatively high level in facilities F1 and F3 and was rarely found in environmental samples of F2.

Since PICRUSt analyses were conducted on data obtained using Greengenes database, we compared the relative abundances of bacterial families determined based on Greengenes database with those determined based on SILVA database (Additional file 1: Table S6). The results showed that the differences in relative abundances were insignificant when all taxa identified using SILVA database were considered (*p* = 0.975). Further, separate comparison of relative abundances of taxa with high relative abundance (> 0.0001) and low relative abundance (< 0.0001) confirmed insignificant differences (*p* = 0.9675 and *p* = 0.1389, respectively) among results obtained based on Greengenes and SILVA databases.

### Network analysis of microbiota showed co-occurrence patterns between Pseudomonadaceae, Enterobacteriaceae, and Rhizobiaceae

**Fig. 8:**
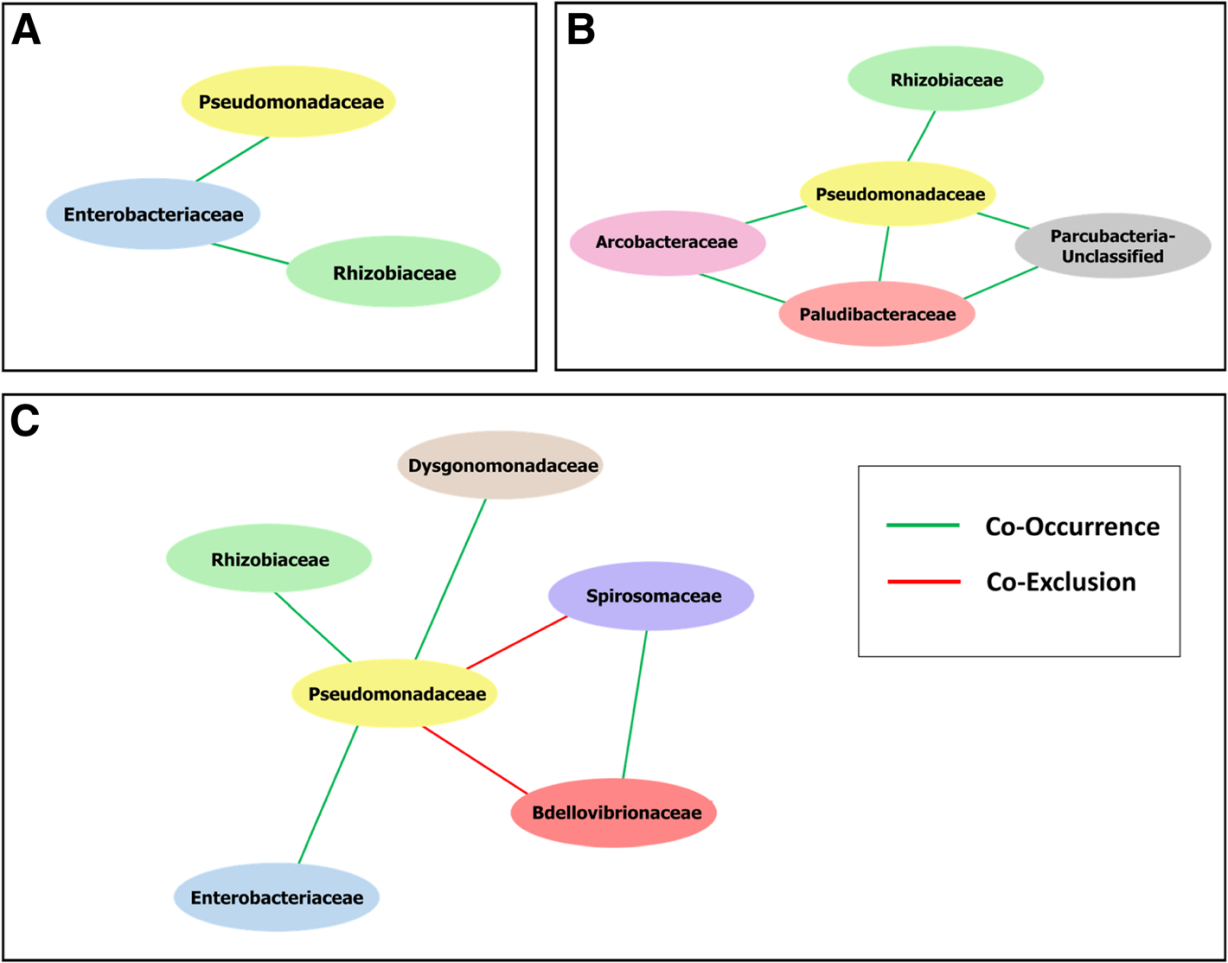
Microbial networks indicating co-occurrence of microbial families in samples collected from three facilities F1, F2, and F3. Green edges represent positive relationship (co-occurrence) among families, whereas the red edges represent negative relationship (co-exclusion) between two connected nodes. Interaction network was sub-grouped with focus on Pseudomonadaceae, a bacterial family that predominated in microbiota of facility F2 that had the highest occurrence of *L*. *monocytogenes*. Interaction correlation between Pseudomonadaceae when only facility F1 samples were analyzed is shown in a panel (**a**). Interaction correlation between Pseudomonadaceae when only facility F3 samples were analyzed is shown in a panel (**b**). Interaction correlation between Pseudomonadaceae and other families when all samples are analyzed together is shown in a panel (**c**)

In order to further explore the relationships between different microbial taxa, especially those that were highly abundant in F2, network analysis was carried out to investigate the co-occurrence or co-exclusion relationship of microbiota in tree fruit packing house built environment. Since facility F2 was previously identified as having the highest occurrence of *L*. *monocytogenes*, as well as having a relatively higher abundance of bacterial family Pseudomonadaceae, we sub-grouped the network to assess the interactions with a focus on the microbial families in direct relationship with Pseudomonadaceae. Families that co-occurred or co-excluded with Pseudomonadaceae were analyzed with all samples combined as well as with just samples from individual facilities. Both analyses were carried out to minimize potentially spurious relationships induced due to the significantly different composition of microbiota found among facilities. In facility F1, Pseudomonadaceae co-occurred only with Enterobacteriaceae, and Enterobacteriaceae was positively correlated with the occurrence of Rhizobiaceae (Fig. 8a). No co-occurrence or co-exclusion relationships between Pseudomonadaceae and other taxa have been found when samples from facility F2 were analyzed separately from other samples. Families Rhizobiaceae, Arcobacteraceae, Paludibacteraceae, and one Parcubacteria unclassified family were found to co-occur with Pseudomonadaceae in samples from facility F3 (Fig. 8b). A similar trend of the relationship was observed when samples from all facilities were analyzed together. In the network of all analyzed samples, Pseudomonadaceae was shown to have a significantly positive correlation with families Rhizobiaceae, Enterobacteriaceae, and Dysgonomonadace. On the other hand, Pseudomonadaceae had co-exclusionary relation with Spirosomaceae and Bdellovibrionaceae (Fig. 8c).

### PICRUSt predicted the differential abundance of bacterial taxa associated with certain functional categories in microbiota found in facility F2 compared to those found in facilities F1 and F3

**Fig. 9:**
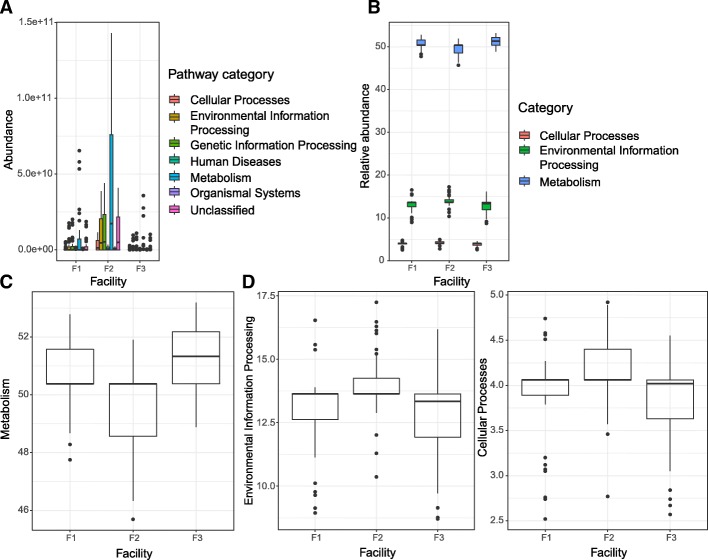
Predicted functional profiles of microbiota in facilities F1, F2, and F3 derived from PICRUSt inference based on tier 1 KEGG ortholog categories (KO). Absolute abundance of tier 1 functional categories by facility is shown in a panel (**a**). Relative abundance of selected tier 1 functional categories by facility is shown in a panel (**b**). Relative abundance of predicted metabolism of microbiota by facility is shown in a panel (**c**). Relative abundance of *Environmental information processing* functional category in microbiota from three facilities is shown in a panel (**d**). Relative abundance of *Cellular processes* functional category in microbiota from three facilities is shown in a panel (**e**)

PICRUSt analysis was performed for functional profile predictions of microbiota based on 16S rRNA marker gene. Firstly, the absolute KEGG ortholog categories (KO) abundance in tier 1 functional categories were plotted for samples grouped by individual facility (Fig. 9a). Facility 2 microbiota samples showed a substantially higher abundance of identified KO in every category, compared to microbiota found in samples from facilities F1 and F3. We further calculated the relative abundance of tier 1 categories of the total absolute abundance in each facility (Fig. 9b). Since categories *metabolism*, *environmental information processing*, and *cellular processes* were considered to be more relevant to our environmental samples, we plotted each of these three categories separately. Microbiota showed a significant difference in functional category *metabolism* when F2 and F1 (*p* = 0.0016) as well as F2 and F3 (*p* = 8.1 × 10^−8^) were compared. There was no significant difference detected in the relative abundance of *metabolism* between F1 and F3 (*p* = 0.0522) (Fig. 9c). Comparing the *environmental information processing* profile, facility F2 again showed a significantly higher relative abundance compared to F1 (*p* = 0.0194) and F3 (*p* = 0.0019). When *cellular processes* profile was compared in microbiota from different facilities, facility 2 KO abundance in this category was significantly different from that in F1 (*p* = 0.0279) and F3 (*p* = 0.0016). The abundance of *cellular processes* KO in facilities F1 and F3 was not significantly different (*p* = 1.000).

## Discussion

### Less sanitary conditions likely contributed to a higher occurrence of *L*. *monocytogenes* in facility F2

Facility F2 had a significantly higher occurrence of *L*. *monocytogenes* compared to facilities F1 and F3. This may be explained by a different facility equipment set-up. For example, while both F1 and F3 were fully equipped, facility F2 did not install a dripping pan under the wet processing line to contain water and organic residues, including leaves, stems, and fruit pieces during processing. Water and organic residues therefore accumulated on the concrete floor underneath the conveyor where they potentially created a nutrient-rich environment suitable for microbial growth. The lack of a proper drainage system and poor cleaning and sanitizing practices may also have contributed to the unique microbiota in the built environment of the facility F2. The presence of cracks in the floor and between the equipment-floor junctions adds on the difficulties of cleaning, and it may promote the growth of microbial biofilm and persistence of *L*. *monocytogenes* contamination in these areas [52].

### Reduced diversity of microbial communities in facility F2 is indicative of the persistence of *L*. *monocytogenes*

Rarefaction curves indicated the lowest bacterial and fungal species richness in environmental samples from facility F2, while microbial richness was substantially higher in facilities F1 and F3. The microbiota and mycobiota rarefaction curves for samples collected in facilities F1 and F3 did not reach a plateau, indicating a considerable undiscovered diversity at a used sequencing depth. The indications of rarefaction curves were confirmed by low alpha diversity in samples from facility F2. Lower diversity in microbial species may result in less competition for nutrients among microorganisms as well as fewer inhibitory secondary metabolites produced by certain microorganisms [53]. Leriche and Carpentier found that a decreased adhesion was found for *L*. *monocytogenes* when it was co-cultured with *Staphylococcus sciuri* due to competition for nutrients [19]. Secondary metabolites, including bacteriocin-like compounds produced by isolated lactic acid bacteria, have also been shown to have antagonistic activity against *L*. *innocua*, a close relative which is commonly used to model the behavior of *L*. *monocytogenes* [54]. Production of enzymes that are lytic to cell wall is another mode of antimicrobial action found in yeast, including *Candida guilliermondii* and *Candida oleophila* that have been shown to secrete hydrolytic enzymes that can be used to control *Botrytis cinerea* on tomato plants [55]. Lower alpha diversity in F2, as well as distinct clustering of microbiota from F2 based on the beta diversity, indicates the ecologically distinct character of facility F2 that potentially supports the growth and/or persistence of *L*. *monocytogenes*. Based on our collected data, it is not possible to speculate whether the lower microbial diversity in facility F2 led to the persistence of *L*. *monocytogenes*, or the establishment of *L*. *monocytogenes* led to reduced alpha diversity and distinct microbial ecology compared to facilities F1 and F3. It is noteworthy, though, that facilities F1 and F3 both have a similar microbiota composition despite different locations.

### Pseudomonadaceae and Dipodascaceae predominate in facility F2 and represent microorganisms that are known biofilm formers and indicators of unhygienic conditions, respectively

When comparing the identified bacterial families and their corresponding relative abundances obtained based on Greengenes database and SILVA database, inconsistencies were mainly found among relatively low abundant taxa. However, statistical comparison showed that analysis based on taxonomic classification using different databases did not result in significantly different composition on a family level. Pseudomonadaceae was predominant in the environment of the facility F2 with persistent *L*. *monocytogenes* contamination and is comprised of known biofilm formers, including *Azomonas*, *Azorhizophilus*, *Azotobacter*, *Mesophilobacter*, and *Pseudomonas* [56]. *Pseudomonas* species are known for their ability to adapt and thrive in the food processing environment [57]. They may have a growth advantage in a cold and humid food processing environment that allows them to outcompete a number of other microorganisms because they can grow on a variety of nutrient sources, tolerate low temperatures, and form a biofilm [58]. These properties make *Pseudomonas* successful in colonization of environmental surfaces. Previous research has demonstrated that *L*. *monocytogenes* adheres to surfaces substantially better when co-cultured with *Pseudomonas fragi*. Biofilm-forming *Pseudomonas* could shelter *L*. *monocytogenes* and enhance its survivability under recurrent cleaning and sanitizing treatments [59]. This protection is conferred by an EPS that provides a physical barrier that reduces diffusion of chemicals into the core of the biofilm structure, as well as provides nutrients for bacterial growth. *L*. *monocytogenes* can survive for a longer period of time when co-cultured with *P*. *pudida* even with no added nutrients [18]. These previous findings and our observation of significantly higher relative abundance of Pseudomonadaceae in facility F2 led us to hypothesize that biofilm formation may support *L*. *monocytogenes* persistence in this facility. Further studies investigating the mechanisms of microbial interactions in microbiota in F2 are hence warranted.

Another, fungal, family Dipodascaceae was the predominant family detected in facility F2 mycobiota, whereas it was low-abundant or absent in facilities F1 and F3. Dipodascaceae are white filamentous fungi belonging to the order of Saccharomycetales. Microorganisms belonging to this family have been previously shown to have an ability to elevate granular sludge lipid accumulation due to the production of linoleic acid [60]. For example, species *Geotrichum candidum*, referred to as “machinery mould,” is a common species belonging to Dipodascaceae family and is ubiquitously present in the natural environment [61]. It is a plant pathogen that causes post-harvest sour-rot spoilage of fruit and vegetable during storage [61]. *G*. *candidum* can form a white slime on surfaces that have contact with produce residues and can be readily removed with good sanitation practices [62]. Therefore, it has been historically used as a fungal indicator of poor hygienic status in fruit and vegetable processing plants that are associated with inadequate sanitation [63]. Although there is no direct information indicating that Dipodascaceae could promote the survival of *L*. *monocytogenes*, the presence of *G*. *candidum* does indicate the overall poor hygienic status of facility F2. With reduced hygienic status, the risk of foodborne pathogen contamination and colonization in the facility increases [64].

The unhygienic environmental conditions in facility F2 may have shaped the microbial communities in the environment toward a predominance of Pseudomonadaceae and Dipodascaceae families, as well as *L*. *monocytogenes*. Furthermore, inadequate cleaning and sanitizing of the facility presents an increased risk for cross-contamination of produce from the produce processing environment.

Although *L*. *monocytogenes* is not able to grow on apples, the pathogen can survive on the surface of the fresh fruit [65]. Given that apples are consumed raw, their contamination with *L*. *monocytogenes* presents a health risk for certain groups of consumers, such as elderly and immunocompromised that can get sick by ingestion of as few as 0.1 million to 10 million CFU of *L*. *monocytogenes*, based on the risk assessment of *L*. *monocytogenes* in Canada [66]. FAO/WHO estimated the risk of *L*. *monocytogenes* based on epidemiological data later in 2014, and predicted that exposure to 10,000 *L*. *monocytogenes* cells results in one case of listeriosis infection in every 20 million people [67]. However, based on two recent outbreaks in the USA and Europe, which involved immunocompromised patients and possibly hypervirulent strains, the probability of infection after consumption of one cell of *L*. *monocytogenes* was estimated to be almost 100,000 times higher than that estimated by FAO/WHO in 2004 based on epidemiologic data of patients from all susceptible population groups [68].

In cases when fresh apples are coated with caramel and punctured with a wooden stick, the risk increases, as this creates microenvironments suitable for pathogen growth [9]. Better understanding of the interactions, growth, and/or persistence of *L*. *monocytogenes* in natural microbiomes, including microbiome biofilms, is needed. Improved knowledge in this area could lay a foundation for precise optimization of cleaning and sanitizing procedures to effectively reduce *L*. *monocytogenes* in built environments through manipulation of microbial interactions.

### Co-occurrence and co-exclusion relationships between Pseudomonadaceae and other bacterial families

We applied network analysis to describe the co-occurrence and co-exclusion relationships between Pseudomonadaceae and other bacterial families that may contribute to *L*. *monocytogenes* survival and persistence in apple packing house environments. The co-occurrence of Enterobacteriaceae, Pseudomonas, and Rhizobiaceae in the environment of the facility 2 is noteworthy, as they are related due to the ease of genetic material exchange [69]. For example, an antibiotic-resistant R factor is inter-transferable among genera comprising Enterobacteriaceae, Pseudomonas, and Rhizobiaceae [70]. Another study based on Adansonian analysis has shown that members of Rhizobiaceae are highly related to strains in *Escherichia coli* and *Enterobacter aerogenes*, while genera *Rhizobium* and *Phytomyxa* from the family of Rhizobiaceae are closely related to members in Pseudomonadaceae family [71]. Besides genetic relatedness, mutualism based on metabolites has also been found between *Pseudomonas aeruginosa* PA14 and *Enterobacter aerogenes*, where the co-culture of the two organisms had at least 14-fold increase in current density compared to either species alone [72]. In contrast, Pseudomonadaceae and Bdellovibrionaceae were found to co-exclude each other when all samples were analyzed together. *Bdellovibrio*, a genus from the family of Bdellovibrionaceae that was shown to negatively co-occur with Pseudomonadaceae in our study, has been previously shown to have predatory activity toward Gram-negative bacteria, especially *Escherichia coli* and *Pseudomonas* (i.e., *Pseudomonas syringae* [73] and *Pseudomonas aeruginosa* [74]). Furthermore, *Bdellovibrio bacteriovorus* has been shown to reduce existing *E*. *coli* and *P*. *fluorescence* biofilm biomass, and *Bdellovibrio*-treated biofilm has been more easily washed off compared to a control [75]. *Pseudomonas aeruginosa* is also considered as one of *B*. *bacteriovorus*’ prey [76]. Limited information is available about characteristics of microorganisms belonging to the family Spriosomaceae, another family that was negatively co-occurring with Pseudomonadaceae. Network analysis based on fungal families identified a fungal family Dipodascaceae which was found in high relative abundance in facility F2 as negatively associated with a number of other fungal families, and positively associated only with an undefined fungal family Saccharomycetales-fam-Incertae-sedis (Additional file 1: Figure S1). Given that many fungal OTUs were classified as unknown and were hence not further classified into known fungal families, these results need to be interpreted with caution. Network analysis of fungal and bacterial families combined showed similar relationship when they were analyzed separately, where bacterial family Pseudomonadaceae was identified as positively associated with the occurrence of Enterobacteriaceae, Rhizobiaceae, and Dysgonomonadaceae, and fungal family Dipodascaceae was found to be positively associated with the presence of “Saccharomycetales-fam-Incertae-sedis,” and negatively associated with the occurrence of “unclassified-Ciliophora” (Additional file 1: Figure S2). Our network analysis indicates a potential mechanism underlying the high prevalence of certain bacterial families in the processing environments. The positive relationship may indicate the mutualistic interactions, whereas the negative correlation may suggest the inhibitory interactions among microbiota. Nevertheless, these hypothesized correlations need to be further studied experimentally.

### Predicted bacterial functional profiles and putative underlying mechanisms

Samples collected in facility F2 had a substantially higher number of identified KOs compared to samples from F1 and F3, indicating a higher number of functional units were characteristic of microbiota detected in facility F2. This is contradictory to what has been found in other studies where they speculated a decline in functionality in less diverse microbiota communities. However, this may also be a result of urbanization-introduced biotic similarity [77], as some of the other studies have shown that low functionality can also be found in highly diverse bacterial communities, as a result of functional redundancy [78].

Facility F2 microbiota had a higher relative abundance of KO in the *metabolism* and *cellular processes* category, whereas it had lower relative KO in the *environmental information processing* category compared to the microbiota from facilities F1 and F3. This is consistent with our hypothesis that led to further analyses of sub-tier categories. Category *metabolism* includes the production of secondary metabolites, which we proposed earlier as one of the potential mechanisms through which persistent colonization of *L*. *monocytogenes* is inhibited in facilities F1 and F3. On the other hand, the bacterial secretion system which belongs to tier 1 category *environmental information processing*, plays a crucial role in the *P*. *aeruginosa* biofilm formation [79]. Cell communication, as one of the sub-categories of *cellular processes*, may also help explain the presence of the biofilm-formers in facility F2 since quorum sensing and bacterial biofilm formation are highly linked [80]. Moreover, previous studies have shown that type VI secretion systems (T6SSs) may be involved in the biofilm formation in Gram-negative bacteria including *Pseudomonas* spp. [81]. Although a clear association between the diversity and functionality of native microorganisms present in the built tree fruit processing facility environment needs to be further investigated to validate these functional predictions, our results provide foundational information that may be used to develop targeted study designs to help elucidate the impact of built environment microbial communities on the survival, growth, and persistence of pathogenic microorganisms such as *L*. *monocytogenes* in food processing facilities. This is particularly important as the use of new biocontrol products is gaining in popularity and needs to be carefully assessed prior to application to avoid adverse outcomes.

### Outlook, novelty, and importance

When designing the cleaning and sanitizing protocols, it is important to consider the microbial landscape in the built environment of food processing facilities. It has been shown that planktonic *L*. *monocytogenes* could be reduced by 5 logs within 30 s of treatment, whereas the only weak effect was observed in attached cells even after an extended treatment time [82]. The complexity of in-house microbiota community is likely to reduce the effectiveness of disinfectants, and bacteria such as Pseudomonadaceae may help foodborne pathogen such as *L*. *monocytogenes* in survival and persistence in food processing environments and thus increase the food safety and public health risk. Our longitudinal survey of tree fruit packinghouse environmental microbiota suggests that the composition and diversity of microorganism found in food processing environments is associated with persisting *L*. *monocytogenes* contamination. Our study provides a data baseline needed for further in-depth investigation of microbial interactions between non-pathogenic and pathogenic microorganisms found in food processing environments. Further research in the area may lead to the optimization of pathogen control strategies and the development of novel biocontrol methods to complement physical and chemical interventions and improve food safety.

## Additional file

Additional file 1: **Table S1.** Metadata for collected samples. **Table S2.** Chi-square test of *L. monocytogenes* occurrence among processing sections. **Table S3.** Chi-square test of *L. monocytogenes* occurrence among facilities. **Table S4.** Results of pairwise PERMANOVA analyses for microbial communities. **Table S5.** Results of pairwise PERMANOVA analyses for fungal communities. **Table S6**. Comparison of relative abundances of bacterial families identified using Greengenes and SILVA database. **Figure S1**. Microbial network indicating co-occurrence of fungal families identified in samples collected from all three facilities. **Figure S2**. Microbial network indicating co-occurrence of bacterial and fungal families in samples collected from three facilities F1, F2, and F3 combined. **List L1.** Data analyses workflow. (DOCX 487 kb)

## Data Availability

Amplicon sequencing data from this study were deposited in NCBI under BioProject accession number PRJNA527988.

## Abbreviations

ANOVA: Analysis of variance
BAM: Bacteriological analytical manual
BLEB: Buffered *Listeria* enrichment broth
DNA: Deoxyribonucleic acid
FDA: Food and Drug Administration
*iap*: Invasion associated protein
ITS: Internal transcribed spacer
KEGG: Kyoto Encyclopedia of Genes and Genomes
LM: *Listeria monocytogenes*
MPN: Most probable number
OTU: Operational taxonomic unit
PCoA: Principal coordinates analysis
PCR: Polymerase chain reaction
PERMANOVA: Permutational multivariate analysis of variance
PICRUSt: Phylogenetic Investigation of Communities by Reconstruction of Unobserved States
RA: Relative abundance
RNA: Ribonucleic acid
TSAYE: Trypticase soy agar with yeast extract
