# Supplementary material for: The occurrence of Listeria monocytogenes is associated with built environment microbiota in three tree fruit processing facilities
Source: Microbiome. 2019 Aug 21;7:115. doi: 10.1186/s40168-019-0726-2 (PMC6702733; doi:10.1186/s40168-019-0726-2)
Supplement: Supplementary file 1 — Table S1. Metadata for collected samples. Table S2. Chi-square test of L. monocytogenes occurrence among processing sections. Table S3. Chi-square test of L. monocytogenes occurrence among facilities. Table S4. Results of pairwise PERMANOVA analyses for microbial communities. Table S5. Results of pairwise PERMANOVA analyses for fungal communities. Table S6. Comparison of relative abundances of bacterial families identified using Greengenes and SILVA database. Figure S1. Microbial network indicating co-occurrence of fungal families identified in samples collected from all three facilities. Figure S2. Microbial network indicating co-occurrence of bacterial and fungal families in samples collected from three facilities F1, F2, and F3 combined. List L1. Data analyses workflow. (DOCX 487 kb) [file 40168_2019_726_MOESM1_ESM.docx]

**Occurrence of *Listeria monocytogenes* is associated with built environment microbiota in three tree fruit processing facilities**

Xiaoqing Tan^1,2^, Taejung Chung^1,2^, Yi Chen^3^, Dumitru Macarisin^3^, Luke LaBorde^1^, Jasna Kovac^1,2^

**^1^** Department of Food Science, The Pennsylvania State University, University Park, PA 16802, USA

^2^ Microbiome Center, Huck Institute of Life Sciences, The Pennsylvania State University, University Park, PA 16802, USA

^3^ Center for Food Safety and Applied Nutrition, Food and Drug Administration, College Park, MD 20740, USA

**Supplementary Materials**

**Table S1:** Metadata for collected samples

| Bacterial community samples^a^ | Fungal community sample | Facility | Sample collection section | Sample collection date | Week | Month | *L. monocytogenes* detection result |
| --- | --- | --- | --- | --- | --- | --- | --- |
| 0105-1s | 0105-1t | F1 | wash | 5-Jan-18 | W5 | January | + |
| 0105-2s | 0105-2t | F1 | dry | 5-Jan-18 | W5 | January | - |
| 0105-3s | 0105-3t | F1 | wax | 5-Jan-18 | W5 | January | - |
| 0105-4s | 0105-4t | F2 | wash | 5-Jan-18 | W5 | January | + |
| 0105-5s | 0105-5t | F2 | dry | 5-Jan-18 | W5 | January | + |
| 0105-6s | 0105-6t | F2 | wax | 5-Jan-18 | W5 | January | + |
| 0105-7s | 0105-7t | F3 | wash | 5-Jan-18 | W5 | January | + |
| 0105-8s | 0105-8t | F3 | dry | 5-Jan-18 | W5 | January | - |
| 0105-9s | 0105-9t | F3 | wax | 5-Jan-18 | W5 | January | + |
| 0119-1s | 0119-1t | F1 | wash | 19-Jan-18 | W6 | January | - |
| 0119-2s | 0119-2t | F1 | dry | 19-Jan-18 | W6 | January | - |
| 0119-3s | 0119-3t | F1 | wax | 19-Jan-18 | W6 | January | + |
| 0119-4s | 0119-4t | F2 | wash | 19-Jan-18 | W6 | January | + |
| 0119-5s | 0119-5t | F2 | dry | 19-Jan-18 | W6 | January | + |
| 0119-6s | 0119-6t | F2 | wax | 19-Jan-18 | W6 | January | + |
| 0119-7s | 0119-7t | F3 | wash | 19-Jan-18 | W6 | January | + |
| 0119-8s | 0119-8t | F3 | dry | 19-Jan-18 | W6 | January | - |
| 0119-9s | 0119-9t | F3 | wax | 19-Jan-18 | W6 | January | - |
| 0202-1s | 0202-1t | F1 | wash | 2-Feb-18 | W7 | February | + |
| 0202-2s | 0202-2t | F1 | dry | 2-Feb-18 | W7 | February | + |
| 0202-3s | 0202-3t | F1 | wax | 2-Feb-18 | W7 | February | - |
| 0202-4s | 0202-4t | F2 | wash | 2-Feb-18 | W7 | February | + |
| 0202-5s | 0202-5t | F2 | dry | 2-Feb-18 | W7 | February | + |
| 0202-6s | 0202-6t | F2 | wax | 2-Feb-18 | W7 | February | + |
| 0202-7s | 0202-7t | F3 | wash | 2-Feb-18 | W7 | February | + |
| 0202-8s | 0202-8t | F3 | dry | 2-Feb-18 | W7 | February | - |
| 0202-9s | 0202-9t | F3 | wax | 2-Feb-18 | W7 | February | - |
| 0216-1s | 0216-1t | F1 | wash | 16-Feb-18 | W8 | February | + |
| 0216-2s | 0216-2t | F1 | dry | 16-Feb-18 | W8 | February | - |
| 0216-3s | 0216-3t | F1 | wax | 16-Feb-18 | W8 | February | - |
| 0216-4s | 0216-4t | F2 | wash | 16-Feb-18 | W8 | February | + |
| 0216-5s | 0216-5t | F2 | dry | 16-Feb-18 | W8 | February | + |
| 0216-6s | 0216-6t | F2 | wax | 16-Feb-18 | W8 | February | + |
| 0216-7s | 0216-7t | F3 | wash | 16-Feb-18 | W8 | February | - |
| 0216-8s | 0216-8t | F3 | dry | 16-Feb-18 | W8 | February | - |
| 0216-9s | 0216-9t | F3 | wax | 16-Feb-18 | W8 | February | - |
| 0302-1s | 0302-1t | F1 | wash | 2-Mar-18 | W9 | March | - |
| 0302-2s | 0302-2t | F1 | dry | 2-Mar-18 | W9 | March | - |
| 0302-3s | 0302-3t | F1 | wax | 2-Mar-18 | W9 | March | - |
| 0302-4s | 0302-4t | F2 | wash | 2-Mar-18 | W9 | March | + |
| 0302-5s | 0302-5t | F2 | dry | 2-Mar-18 | W9 | March | + |
| 0302-6s | 0302-6t | F2 | wax | 2-Mar-18 | W9 | March | + |
| 0302-7s | 0302-7t | F3 | wash | 2-Mar-18 | W9 | March | + |
| 0302-8s | 0302-8t | F3 | dry | 2-Mar-18 | W9 | March | - |
| 0302-9s | 0302-9t | F3 | wax | 2-Mar-18 | W9 | March | + |
| 0316-1s | 0316-1t | F1 | wash | 16-Mar-18 | W10 | March | - |
| 0316-2s | 0316-2t | F1 | dry | 16-Mar-18 | W10 | March | - |
| 0316-3s | 0316-3t | F1 | wax | 16-Mar-18 | W10 | March | - |
| 0316-4s | 0316-4t | F2 | wash | 16-Mar-18 | W10 | March | + |
| 0316-5s | 0316-5t | F2 | dry | 16-Mar-18 | W10 | March | + |
| 0316-6s | 0316-6t | F2 | wax | 16-Mar-18 | W10 | March | + |
| 0316-7s | 0316-7t | F3 | wash | 16-Mar-18 | W10 | March | + |
| 0316-8s | 0316-8t | F3 | dry | 16-Mar-18 | W10 | March | + |
| 0316-9s | 0316-9t | F3 | wax | 16-Mar-18 | W10 | March | - |
| 0404-1s | 0404-1t | F1 | wash | 4-Apr-18 | W11 | April | - |
| 0404-2s | 0404-2t | F1 | dry | 4-Apr-18 | W11 | April | - |
| 0404-3s | 0404-3t | F1 | wax | 4-Apr-18 | W11 | April | - |
| 0404-4s | 0404-4t | F2 | wash | 4-Apr-18 | W11 | April | + |
| 0404-5s | 0404-5t | F2 | dry | 4-Apr-18 | W11 | April | + |
| 0404-6s | 0404-6t | F2 | wax | 4-Apr-18 | W11 | April | + |
| 0404-7s | 0404-7t | F3 | wash | 4-Apr-18 | W11 | April | - |
| 0404-8s | 0404-8t | F3 | dry | 4-Apr-18 | W11 | April | - |
| 0404-9s | 0404-9t | F3 | wax | 4-Apr-18 | W11 | April | - |
| 0416-1s | 0416-1t | F1 | wash | 16-Apr-18 | W12 | April | - |
| 0416-2s | 0416-2t | F1 | dry | 16-Apr-18 | W12 | April | - |
| 0416-3s | 0416-3t | F1 | wax | 16-Apr-18 | W12 | April | - |
| 0416-4s | 0416-4t | F2 | wash | 16-Apr-18 | W12 | April | + |
| 0416-5s | 0416-5t | F2 | dry | 16-Apr-18 | W12 | April | + |
| 0416-6s | 0416-6t | F2 | wax | 16-Apr-18 | W12 | April | + |
| 0416-7s | 0416-7t | F3 | wash | 16-Apr-18 | W12 | April | - |
| 0416-8s | 0416-8t | F3 | dry | 16-Apr-18 | W12 | April | - |
| 0416-9s | 0416-9t | F3 | wax | 16-Apr-18 | W12 | April | - |
| 0427-1s | 0427-1t | F1 | wash | 27-Apr-18 | W13 | April | + |
| 0427-2s | 0427-2t | F1 | dry | 27-Apr-18 | W13 | April | + |
| 0427-3s | 0427-3t | F1 | wax | 27-Apr-18 | W13 | April | - |
| 0427-4s | 0427-4t | F2 | wash | 27-Apr-18 | W13 | April | + |
| 0427-5s | 0427-5t | F2 | dry | 27-Apr-18 | W13 | April | + |
| 0427-6s | 0427-6t | F2 | wax | 27-Apr-18 | W13 | April | + |
| 0427-7s | 0427-7t | F3 | wash | 27-Apr-18 | W13 | April | - |
| 0427-8s | 0427-8t | F3 | dry | 27-Apr-18 | W13 | April | - |
| 0427-9s | 0427-9t | F3 | wax | 27-Apr-18 | W13 | April | - |
| 1103-1s | 1103-1t | F1 | wash | 3-Nov-17 | W1 | November | + |
| 1103-2s | 1103-2t | F1 | dry | 3-Nov-17 | W1 | November | - |
| 1103-3s | 1103-3t | F1 | wax | 3-Nov-17 | W1 | November | - |
| 1103-4s | 1103-4t | F2 | wash | 3-Nov-17 | W1 | November | + |
| 1103-5s | 1103-5t | F2 | dry | 3-Nov-17 | W1 | November | + |
| 1103-6s | 1103-6t | F2 | wax | 3-Nov-17 | W1 | November | + |
| 1103-7s | 1103-7t | F3 | wash | 3-Nov-17 | W1 | November | - |
| 1103-8s | 1103-8t | F3 | dry | 3-Nov-17 | W1 | November | + |
| 1103-9s | 1103-9t | F3 | wax | 3-Nov-17 | W1 | November | - |
| 1121-1s | 1121-1t | F1 | wash | 21-Nov-17 | W2 | November | + |
| 1121-2s | 1121-2t | F1 | dry | 21-Nov-17 | W2 | November | - |
| 1121-3s | 1121-3t | F1 | wax | 21-Nov-17 | W2 | November | - |
| 1121-4s | 1121-4t | F2 | wash | 21-Nov-17 | W2 | November | + |
| 1121-5s | 1121-5t | F2 | dry | 21-Nov-17 | W2 | November | + |
| 1121-6s | 1121-6t | F2 | wax | 21-Nov-17 | W2 | November | + |
| 1121-7s | 1121-7t | F3 | wash | 21-Nov-17 | W2 | November | + |
| 1121-8s | 1121-8t | F3 | dry | 21-Nov-17 | W2 | November | + |
| 1121-9s | 1121-9t | F3 | wax | 21-Nov-17 | W2 | November | + |
| 1208-1s | 1208-1t | F1 | wash | 8-Dec-17 | W3 | December | + |
| 1208-2s | 1208-2t | F1 | dry | 8-Dec-17 | W3 | December | - |
| 1208-3s | 1208-3t | F1 | wax | 8-Dec-17 | W3 | December | - |
| 1208-4s | 1208-4t | F2 | wash | 8-Dec-17 | W3 | December | + |
| 1208-5s | 1208-5t | F2 | dry | 8-Dec-17 | W3 | December | + |
| 1208-6s | 1208-6t | F2 | wax | 8-Dec-17 | W3 | December | + |
| 1208-7s | 1208-7t | F3 | wash | 8-Dec-17 | W3 | December | + |
| 1208-8s | 1208-8t | F3 | dry | 8-Dec-17 | W3 | December | + |
| 1208-9s | 1208-9t | F3 | wax | 8-Dec-17 | W3 | December | + |
| 1218-1s | 1218-1t | F1 | wash | 18-Dec-17 | W4 | December | - |
| 1218-2s | 1218-2t | F1 | dry | 18-Dec-17 | W4 | December | + |
| 1218-3s | 1218-3t | F1 | wax | 18-Dec-17 | W4 | December | - |
| 1218-4s | 1218-4t | F2 | wash | 18-Dec-17 | W4 | December | + |
| 1218-5s | 1218-5t | F2 | dry | 18-Dec-17 | W4 | December | + |
| 1218-6s | 1218-6t | F2 | wax | 18-Dec-17 | W4 | December | + |
| 1218-7s | 1218-7t | F3 | wash | 18-Dec-17 | W4 | December | - |
| 1218-8s | 1218-8t | F3 | dry | 18-Dec-17 | W4 | December | + |
| 1218-9s | 1218-9t | F3 | wax | 18-Dec-17 | W4 | December | - |

^a^Amplicon sequences corresponding to listed samples are available on NCBI under BioProject accession number PRJNA527988.

**Table S2:** Chi-square test of *L. monocytogenes* occurrence among processing sections

| *L. monocytogenes* occurrence | Section^a^ | | |
| --- | --- | --- | --- |
|  | Dry | Wash | Wax |
| Absent | 18 | 12 | 21 |
| Present | 21 | 27 | 18 |
|  | Chi-Square | DF | P-value |
| Pearson | 4.38 | 2 | 0.112 |
| Likelihood | 4.454 | 2 | 0.108 |

^a^DF, degree of freedom.

In Facility F1 there was a significant difference among occurrence of *L. monocytogenes* in different sampled sections (P = 0.029). No significant difference in *L. monocytogenes* occurrence was observed among samples collected from different sections in Facility F3 (P = 0.476). Statistical analysis was not conducted for samples collected in facility F2 given that all samples from Facility F2 were positive for *L. monocytogenes*.

**Table S3:** Chi-square test of *L. monocytogenes* occurrence among facilities

| *L. monocytogenes* occurrence | Facility^a^ | | |
| --- | --- | --- | --- |
|  | F1 | F2 | F3 |
| Absent | 28 | 0 | 23 |
| Present | 11 | 39 | 16 |
|  | Chi-Square | DF | P-value |
| Pearson | 46.51 | 2 | < 0.001 |
| Likelihood | 61.07 | 2 | < 0.001 |

^a^DF, degree of freedom.

**Table S4:** Results of pairwise PERMANOVA analyses for microbial communities

| Factor | DF^a^ | Sums of squares | F model | R2^b^ | P value | P adjusted |
| --- | --- | --- | --- | --- | --- | --- |
| January vs February | 1 | 0.431 | 1.052 | 0.030 | 0.347 | 1.000 |
| January vs March | 1 | 0.524 | 1.307 | 0.037 | 0.083 | 1.000 |
| January vs April | 1 | 0.559 | 1.397 | 0.031 | 0.040 | 0.600 |
| January vs November | 1 | 0.534 | 1.296 | 0.037 | 0.044 | 0.660 |
| January vs December | 1 | 0.330 | 0.783 | 0.023 | 0.930 | 1.000 |
| February vs March | 1 | 0.310 | 0.785 | 0.023 | 0.861 | 1.000 |
| February vs April | 1 | 0.571 | 1.441 | 0.032 | 0.030 | 0.450 |
| February vs November | 1 | 0.549 | 1.349 | 0.038 | 0.050 | 0.750 |
| February vs December | 1 | 0.447 | 1.074 | 0.031 | 0.299 | 1.000 |
| March vs April | 1 | 0.549 | 1.411 | 0.032 | 0.055 | 0.825 |
| March vs November | 1 | 0.588 | 1.477 | 0.042 | 0.025 | 0.375 |
| March vs December | 1 | 0.553 | 1.359 | 0.038 | 0.043 | 0.645 |
| April vs November | 1 | 0.651 | 1.632 | 0.037 | 0.004 | 0.060 |
| April vs December | 1 | 0.675 | 1.665 | 0.037 | 0.005 | 0.075 |
| November vs December | 1 | 0.442 | 1.056 | 0.030 | 0.330 | 1.000 |
| F1 vs F2 | 1 | 2.788 | 7.664 | 0.092 | 0.001 | 0.003 |
| F1 vs F3 | 1 | 1.525 | 3.904 | 0.049 | 0.001 | 0.003 |
| F2 vs F3 | 1 | 3.259 | 8.983 | 0.106 | 0.001 | 0.003 |
| Wash vs dry | 1 | 0.713 | 1.780 | 0.023 | 0.003 | 0.009 |
| Wash vs wax | 1 | 1.346 | 3.337 | 0.042 | 0.001 | 0.003 |
| Dry vs wax | 1 | 0.716 | 1.801 | 0.023 | 0.003 | 0.009 |

^a^DF, degree of freedom.
^b^R2, R square.

**Table S5:** Results of pairwise PERMANOVA analyses for fungal communities

| Factor | DF^a^ | Sums of squares | F model | R2b | P value | P adjusted |
| --- | --- | --- | --- | --- | --- | --- |
| January vs February | 1 | 0.152 | 0.688 | 0.020 | 0.588 | 1.000 |
| January vs March | 1 | 0.195 | 0.857 | 0.025 | 0.474 | 1.000 |
| January vs April | 1 | 0.436 | 1.714 | 0.038 | 0.138 | 1.000 |
| January vs November | 1 | 0.303 | 1.261 | 0.036 | 0.215 | 1.000 |
| January vs December | 1 | 0.163 | 0.734 | 0.021 | 0.514 | 1.000 |
| February vs March | 1 | 0.212 | 0.886 | 0.025 | 0.454 | 1.000 |
| February vs April | 1 | 0.234 | 0.892 | 0.020 | 0.454 | 1.000 |
| February vs November | 1 | 0.382 | 1.521 | 0.043 | 0.163 | 1.000 |
| February vs December | 1 | 0.165 | 0.712 | 0.020 | 0.570 | 1.000 |
| March vs April | 1 | 0.247 | 0.919 | 0.021 | 0.462 | 1.000 |
| March vs November | 1 | 0.532 | 2.053 | 0.057 | 0.078 | 1.000 |
| March vs December | 1 | 0.459 | 1.913 | 0.053 | 0.108 | 1.000 |
| April vs November | 1 | 0.522 | 1.871 | 0.042 | 0.100 | 1.000 |
| April vs December | 1 | 0.335 | 1.270 | 0.029 | 0.236 | 1.000 |
| November vs December | 1 | 0.172 | 0.680 | 0.020 | 0.599 | 1.000 |
| F1 vs F2 | 1 | 10.111 | 82.072 | 0.519 | 0.001 | 0.003 |
| F1 vs F3 | 1 | 3.247 | 23.382 | 0.235 | 0.001 | 0.003 |
| F2 vs F3 | 1 | 8.281 | 65.764 | 0.464 | 0.001 | 0.003 |
| Wash vs dry | 1 | 0.748 | 3.175 | 0.040 | 0.019 | 0.057 |
| Wash vs wax | 1 | 1.167 | 4.874 | 0.060 | 0.004 | 0.012 |
| Dry vs wax | 1 | 0.243 | 0.951 | 0.012 | 0.412 | 1.000 |

^a^DF, degree of freedom.
^b^R2, R square.

**Table S6**: Comparison of relative abundances of bacterial families identified using Greengenes and SILVA database. Statistical t test indicated insignificant differences in relative abundances of bacterial families obtained using these two databases.

|  | Relative abundance | |
| --- | --- | --- |
| Bacterial family | Greengenes | SILVA |
| Pseudomonadaceae | 21.95321 | 21.79674 |
| Flavobacteriaceae | 19.30667 | 18.83549 |
| Weeksellaceae | 11.53586 | 10.65667 |
| Moraxellaceae | 8.59841 | 8.54560 |
| Burkholderiaceae | 7.12522 | 7.07451 |
| Sphingomonadaceae | 6.04874 | 6.01967 |
| Caulobacteraceae | 5.69444 | 5.64196 |
| Xanthomonadaceae | 5.61480 | 5.58256 |
| Sphingobacteriaceae | 4.02314 | 3.96145 |
| Enterobacteriaceae | 2.49579 | 2.48440 |
| Rhizobiaceae | 2.42454 | 2.39619 |
| Rhodobacteraceae | 2.25574 | 2.25949 |
| Chitinophagaceae | 2.14125 | 2.06358 |
| Arcobacteraceae | 1.38955 | 1.39003 |
| Azospirillaceae | 1.32850 | 1.33916 |
| Bdellovibrionaceae | 1.18188 | 1.17862 |
| Microbacteriaceae | 1.06613 | 1.06179 |
| env.OPS_17 | 0.85030 | 0.84204 |
| uncultured | 0.82669 | 0.83509 |
| Mycobacteriaceae | 0.80599 | 0.81442 |
| Beijerinckiaceae | 0.72306 | 0.72184 |
| Spirosomaceae | 0.60753 | 0.61613 |
| Aeromonadaceae | 0.55232 | 0.56030 |
| Blastocatellaceae | 0.43648 | 0.42639 |
| Nocardiaceae | 0.36724 | 0.36777 |
| Propionibacteriaceae | 0.36610 | 0.36678 |
| Rhodanobacteraceae | 0.36084 | 0.35287 |
| Bacteriovoracaceae | 0.31219 | 0.31234 |
| Rhodocyclaceae | 0.30830 | 0.31850 |
| Alphaproteobacteria_unclassified | 0.30386 | 0.30260 |
| Methylophilaceae | 0.29097 | 0.28552 |
| Gammaproteobacteria_unclassified | 0.28070 | 0.27677 |
| Alteromonadaceae | 0.25693 | 0.25551 |
| Bacteria_unclassified | 0.25264 | 0.25194 |
| Microscillaceae | 0.24414 | 0.23962 |
| Dysgonomonadaceae | 0.20361 | 0.18259 |
| Acetobacteraceae | 0.19044 | 0.18498 |
| Shewanellaceae | 0.17066 | 0.16014 |
| Rubritaleaceae | 0.14377 | 0.14822 |
| Xanthobacteraceae | 0.13429 | 0.12895 |
| Proteobacteria_unclassified | 0.13272 | 0.13531 |
| Haliangiaceae | 0.13201 | 0.13690 |
| Paludibacteraceae | 0.13179 | 0.13590 |
| SM2D12 | 0.11850 | 0.11782 |
| Devosiaceae | 0.11832 | 0.11762 |
| Hymenobacteraceae | 0.11331 | 0.11484 |
| Polyangiaceae | 0.10762 | 0.10193 |
| Verrucomicrobiaceae | 0.10101 | 0.09299 |
| Opitutaceae | 0.09664 | 0.09477 |
| Leuconostocaceae | 0.09368 | 0.08742 |
| WPS-2_fa | 0.09046 | 0.09040 |
| Micrococcaceae | 0.08059 | 0.08424 |
| Intrasporangiaceae | 0.08058 | 0.07391 |
| Bacteroidaceae | 0.07523 | 0.07570 |
| Nocardioidaceae | 0.07380 | 0.07491 |
| Chthoniobacteraceae | 0.06875 | 0.06378 |
| Crocinitomicaceae | 0.06746 | 0.06815 |
| Obscuribacterales_fa | 0.06604 | 0.06338 |
| Myxococcales_unclassified | 0.06248 | 0.06855 |
| Bacteroidia_unclassified | 0.06223 | 0.06020 |
| Dermatophilaceae | 0.05847 | 0.06120 |
| Betaproteobacteriales_unclassified | 0.05835 | 0.05365 |
| Sericytochromatia_fa | 0.05239 | 0.05365 |
| Unknown_Family | 0.04943 | 0.05047 |
| Thiovulaceae | 0.04654 | 0.04451 |
| 0319-6G20 | 0.04482 | 0.04510 |
| Sulfurospirillaceae | 0.04030 | 0.03954 |
| Hyphomicrobiaceae | 0.03759 | 0.03874 |
| Pirellulaceae | 0.03697 | 0.03676 |
| Cellvibrionaceae | 0.03498 | 0.03696 |
| Methylacidiphilaceae | 0.03480 | 0.03318 |
| Rhizobiales_unclassified | 0.03462 | 0.03219 |
| Xiphinematobacteraceae | 0.03416 | 0.03179 |
| NS11-12_marine_group | 0.03365 | 0.03258 |
| Nakamurellaceae | 0.03102 | 0.03298 |
| Micrococcales_unclassified | 0.03099 | 0.03258 |
| Legionellaceae | 0.03095 | 0.02742 |
| Cytophagaceae | 0.02972 | 0.02821 |
| Oligoflexaceae | 0.02955 | 0.02643 |
| Carnobacteriaceae | 0.02943 | 0.02682 |
| JG30-KF-CM45 | 0.02851 | 0.03060 |
| Deltaproteobacteria_unclassified | 0.02837 | 0.02941 |
| Absconditabacteriales_(SR1)_fa | 0.02832 | 0.02841 |
| A0839 | 0.02808 | 0.02960 |
| Sphingobacteriales_unclassified | 0.02711 | 0.02484 |
| Rhodopirillaceae | 0.02650 | 0.02086 |
| A4b | 0.02636 | 0.02424 |
| Beutenbergiaceae | 0.02489 | 0.02345 |
| Corynebacteriales_unclassified | 0.02395 | 0.02424 |
| KD3-10 | 0.02356 | 0.02523 |
| Saprospiraceae | 0.02310 | 0.02325 |
| Prolixibacteraceae | 0.02103 | 0.02066 |
| JGI_0000069-P22_fa | 0.02022 | 0.02007 |
| Micavibrionales_unclassified | 0.02002 | 0.01888 |
| Aquaspirillaceae | 0.01986 | 0.02126 |
| Magnetospirillaceae | 0.01908 | 0.01927 |
| Cytophagales_unclassified | 0.01892 | 0.01768 |
| Clostridiaceae_1 | 0.01836 | 0.01828 |
| Neisseriaceae | 0.01834 | 0.01788 |
| Fimbriimonadaceae | 0.01715 | 0.01689 |
| Hyphomonadaceae | 0.01712 | 0.01470 |
| WD2101_soil_group | 0.01682 | 0.01570 |
| Gemmataceae | 0.01595 | 0.01431 |
| Terrimicrobiaceae | 0.01591 | 0.01609 |
| Phaselicystidaceae | 0.01568 | 0.01590 |
| Actinobacteria_unclassified | 0.01552 | 0.01629 |
| Fibrobacteraceae | 0.01437 | 0.01291 |
| Paracaedibacteraceae | 0.01382 | 0.01212 |
| Cellulomonadaceae | 0.01378 | 0.01291 |
| Diplorickettsiaceae | 0.01317 | 0.01252 |
| Rhodospirillaceae | 0.01308 | 0.01192 |
| Tannerellaceae | 0.01289 | 0.01172 |
| Saccharimonadales_fa | 0.01275 | 0.01192 |
| Trueperaceae | 0.01255 | 0.01212 |
| Kineosporiaceae | 0.01250 | 0.01291 |
| Dermabacteraceae | 0.01106 | 0.01172 |
| Micromonosporaceae | 0.01098 | 0.00874 |
| Geodermatophilaceae | 0.00969 | 0.01073 |
| Bacteroidales_unclassified | 0.00968 | 0.00815 |
| Methylopilaceae | 0.00953 | 0.00874 |
| Subgroup_6_fa | 0.00915 | 0.01113 |
| Tepidisphaeraceae | 0.00914 | 0.00894 |
| Deinococcaceae | 0.00863 | 0.00815 |
| Actinomycetaceae | 0.00856 | 0.00854 |
| Ruminococcaceae | 0.00842 | 0.00755 |
| Kaistiaceae | 0.00738 | 0.00616 |
| Saccharimonadaceae | 0.00737 | 0.00676 |
| Cyclobacteriaceae | 0.00736 | 0.00874 |
| Geminicoccaceae | 0.00730 | 0.00656 |
| Parachlamydiaceae | 0.00701 | 0.00616 |
| Marinilabiliaceae | 0.00700 | 0.00556 |
| Parcubacteria_unclassified | 0.00689 | 0.00775 |
| Lachnospiraceae | 0.00674 | 0.00636 |
| Aerococcaceae | 0.00662 | 0.00715 |
| P3OB-42 | 0.00655 | 0.00695 |
| Xanthomonadales_unclassified | 0.00623 | 0.00457 |
| Solirubrobacteraceae | 0.00619 | 0.00556 |
| Rhodospirillales_unclassified | 0.00611 | 0.00596 |
| Procabacteriaceae | 0.00607 | 0.00556 |
| Desulfovibrionaceae | 0.00571 | 0.00636 |
| Solibacteraceae_(Subgroup_3) | 0.00568 | 0.00477 |
| Dietziaceae | 0.00526 | 0.00437 |
| Flavobacteriales_unclassified | 0.00523 | 0.00397 |
| Oxyphotobacteria_unclassified | 0.00514 | 0.00576 |
| Rickettsiaceae | 0.00505 | 0.00258 |
| Pleomorphomonadaceae | 0.00495 | 0.00517 |
| Rikenellaceae | 0.00490 | 0.00437 |
| Christensenellaceae | 0.00478 | 0.00338 |
| Geobacteraceae | 0.00471 | 0.00576 |
| Lactobacillales_unclassified | 0.00452 | 0.00417 |
| Brevibacteriaceae | 0.00437 | 0.00457 |
| Nitrosomonadaceae | 0.00417 | 0.00358 |
| Rubinisphaeraceae | 0.00415 | 0.00616 |
| Synergistaceae | 0.00413 | 0.00457 |
| Micavibrionaceae | 0.00407 | 0.00417 |
| 37-13 | 0.00405 | 0.00417 |
| Acidobacteriaceae_(Subgroup_1) | 0.00395 | 0.00238 |
| FBP_fa | 0.00395 | 0.00298 |
| Caedibacteraceae | 0.00392 | 0.00397 |
| Gracilibacteria_fa | 0.00382 | 0.00358 |
| Chloroflexaceae | 0.00379 | 0.00397 |
| Caldilineaceae | 0.00372 | 0.00258 |
| Lentimicrobiaceae | 0.00360 | 0.00238 |
| Elsteraceae | 0.00353 | 0.00358 |
| Tepidisphaerales_unclassified | 0.00348 | 0.00238 |
| Clostridiales_unclassified | 0.00347 | 0.00179 |
| Saccharimonadales_unclassified | 0.00347 | 0.00318 |
| OPB56_fa | 0.00342 | 0.00318 |
| Desulfobulbaceae | 0.00333 | 0.00397 |
| Myxococcaceae | 0.00331 | 0.00397 |
| Gemmatimonadaceae | 0.00329 | 0.00358 |
| Reyranellaceae | 0.00326 | 0.00397 |
| Verrucomicrobiae_unclassified | 0.00322 | 0.00397 |
| Rickettsiales_unclassified | 0.00318 | 0.00219 |
| Gastranaerophilales_fa | 0.00311 | 0.00278 |
| Micrococcales_Incertae_Sedis | 0.00304 | 0.00219 |
| Rhodothermaceae | 0.00302 | 0.00219 |
| Solimonadaceae | 0.00290 | 0.00397 |
| KD4-96_fa | 0.00274 | 0.00397 |
| Pedosphaeraceae | 0.00273 | 0.00199 |
| Ignavibacteria_unclassified | 0.00271 | 0.00298 |
| 67-14 | 0.00266 | 0.00238 |
| Sanguibacteraceae | 0.00264 | 0.00099 |
| Holophagaceae | 0.00260 | 0.00159 |
| Ardenticatenaceae | 0.00259 | 0.00219 |
| PB19_fa | 0.00255 | 0.00358 |
| Micropepsaceae | 0.00252 | 0.00199 |
| Chloroflexi_unclassified | 0.00242 | 0.00179 |
| mle1-27 | 0.00231 | 0.00238 |
| Chlamydiales_unclassified | 0.00231 | 0.00179 |
| Roseiflexaceae | 0.00230 | 0.00318 |
| Veillonellaceae | 0.00230 | 0.00219 |
| SC-I-84 | 0.00226 | 0.00278 |
| Chitinibacteraceae | 0.00218 | 0.00219 |
| Oligoflexales_unclassified | 0.00213 | 0.00139 |
| Halomonadaceae | 0.00212 | 0.00219 |
| Sandaracinaceae | 0.00209 | 0.00199 |
| Kallotenuales_unclassified | 0.00205 | 0.00199 |
| Gaiellaceae | 0.00200 | 0.00159 |
| Demequinaceae | 0.00196 | 0.00199 |
| Deferribacteraceae | 0.00195 | 0.00219 |
| Anaerolineaceae | 0.00178 | 0.00199 |
| SJA-28_fa | 0.00168 | 0.00199 |
| Bacillales_unclassified | 0.00163 | 0.00099 |
| SBR1031_fa | 0.00159 | 0.00119 |
| Herpetosiphonaceae | 0.00159 | 0.00179 |
| Alteromonadales_unclassified | 0.00159 | 0.00179 |
| WCHB1-41_fa | 0.00150 | 0.00159 |
| Streptomycetaceae | 0.00141 | 0.00099 |
| Coxiellaceae | 0.00140 | 0.00119 |
| R7C24_fa | 0.00137 | 0.00219 |
| Acidithiobacillaceae | 0.00135 | 0.00139 |
| Promicromonosporaceae | 0.00134 | 0.00099 |
| Vampirovibrionales_fa | 0.00132 | 0.00179 |
| Bacillaceae | 0.00127 | 0.00159 |
| Chitinophagales_unclassified | 0.00124 | 0.00099 |
| Phycisphaerae_unclassified | 0.00124 | 0.00159 |
| Chroococcidiopsaceae | 0.00122 | 0.00060 |
| 01D2Z36 | 0.00118 | 0.00079 |
| Amoebophilaceae | 0.00113 | 0.00060 |
| CHAB-XI-27_fa | 0.00110 | 0.00060 |
| vadinHA49_fa | 0.00109 | 0.00099 |
| Spirochaetaceae | 0.00109 | 0.00159 |
| Phycisphaeraceae | 0.00107 | 0.00060 |
| Frankiales_unclassified | 0.00107 | 0.00159 |
| Iamiaceae | 0.00104 | 0.00119 |
| UA11_fa | 0.00103 | 0.00119 |
| KD1-131 | 0.00098 | 0.00199 |
| AKYH767 | 0.00096 | 0.00079 |
| Dongiaceae | 0.00095 | 0.00119 |
| Subgroup_6_unclassified | 0.00095 | 0.00079 |
| Ignavibacteriales_unclassified | 0.00092 | 0.00099 |
| Bacteroidetes_vadinHA17 | 0.00090 | 0.00099 |
| Rhizobiales_Incertae_Sedis | 0.00089 | 0.00060 |
| RBG-13-54-9_fa | 0.00089 | 0.00079 |
| Babeliales_unclassified | 0.00087 | 0.00079 |
| Staphylococcaceae | 0.00086 | 0.00040 |
| TK10_fa | 0.00086 | 0.00060 |
| Cyanobacteria_unclassified | 0.00086 | 0.00099 |
| NS9_marine_group | 0.00082 | 0.00099 |
| Prevotellaceae | 0.00082 | 0.00079 |
| M2PB4-65_termite_group | 0.00080 | 0.00099 |
| AKIW781 | 0.00080 | 0.00040 |
| Thermoleophilia_unclassified | 0.00079 | 0.00079 |
| uncultured_fa | 0.00078 | 0.00099 |
| Schlesneriaceae | 0.00076 | 0.00099 |
| Desulfuromonadales_unclassified | 0.00076 | 0.00040 |
| Elusimicrobiaceae | 0.00075 | 0.00079 |
| Lactobacillaceae | 0.00074 | 0.00060 |
| Archangiaceae | 0.00074 | 0.00040 |
| Victivallaceae | 0.00071 | 0.00159 |
| Propionibacteriales_unclassified | 0.00071 | 0.00040 |
| Chitinimonadaceae | 0.00070 | 0.00040 |
| SBR1031_unclassified | 0.00069 | 0.00060 |
| Bifidobacteriaceae | 0.00067 | 0.00000 |
| Chthonomonadaceae | 0.00067 | 0.00060 |
| Puniceicoccaceae | 0.00066 | 0.00139 |
| Steroidobacteraceae | 0.00065 | 0.00079 |
| Pseudomonadales_unclassified | 0.00065 | 0.00079 |
| Isosphaeraceae | 0.00064 | 0.00099 |
| Verrucomicrobiales_unclassified | 0.00063 | 0.00099 |
| Nostocaceae | 0.00063 | 0.00060 |
| Dermacoccaceae | 0.00063 | 0.00000 |
| Planctomycetes_unclassified | 0.00061 | 0.00040 |
| Holosporaceae | 0.00060 | 0.00060 |
| ABY1_unclassified | 0.00059 | 0.00020 |
| OM190_fa | 0.00059 | 0.00000 |
| Nannocystaceae | 0.00059 | 0.00079 |
| Midichloriaceae | 0.00058 | 0.00060 |
| Bernardetiaceae | 0.00057 | 0.00060 |
| Stappiaceae | 0.00056 | 0.00119 |
| Syntrophomonadaceae | 0.00056 | 0.00040 |
| D05-2 | 0.00054 | 0.00020 |
| CPR2_fa | 0.00053 | 0.00020 |
| Parcubacteria_fa | 0.00053 | 0.00020 |
| Blfdi19 | 0.00051 | 0.00020 |
| Caulobacterales_unclassified | 0.00051 | 0.00060 |
| Subgroup_7_fa | 0.00050 | 0.00060 |
| Melainabacteria_unclassified | 0.00049 | 0.00040 |
| KD3-93 | 0.00049 | 0.00139 |
| Pseudonocardiaceae | 0.00048 | 0.00079 |
| Campylobacterales_unclassified | 0.00048 | 0.00020 |
| Thermoanaerobaculaceae | 0.00046 | 0.00079 |
| Gaiellales_unclassified | 0.00046 | 0.00079 |
| Family_XII | 0.00044 | 0.00020 |
| Labraceae | 0.00044 | 0.00040 |
| Simkaniaceae | 0.00043 | 0.00079 |
| Orbaceae | 0.00043 | 0.00060 |
| Planococcaceae | 0.00043 | 0.00119 |
| Lineage_IIb_fa | 0.00042 | 0.00060 |
| BIrii41 | 0.00040 | 0.00099 |
| Phormidiaceae | 0.00040 | 0.00040 |
| Chthoniobacterales_unclassified | 0.00039 | 0.00000 |
| Firmicutes_unclassified | 0.00039 | 0.00020 |
| Candidatus_Falkowbacteria_fa | 0.00037 | 0.00040 |
| Eubacteriaceae | 0.00037 | 0.00000 |
| AB1 | 0.00037 | 0.00040 |
| Nitrincolaceae | 0.00034 | 0.00000 |
| Cryptosporangiaceae | 0.00034 | 0.00060 |
| Parvibaculaceae | 0.00034 | 0.00000 |
| Bacteroidetes_unclassified | 0.00033 | 0.00020 |
| Frankiaceae | 0.00033 | 0.00040 |
| Rokubacteriales_fa | 0.00032 | 0.00020 |
| EV818SWSAP88_fa | 0.00030 | 0.00020 |
| Streptosporangiaceae | 0.00030 | 0.00020 |
| T34 | 0.00029 | 0.00020 |
| SB-5 | 0.00029 | 0.00000 |
| Subgroup_17_fa | 0.00029 | 0.00040 |
| SAR324_clade(Marine_group_B)_fa | 0.00028 | 0.00020 |
| Microtrichaceae | 0.00027 | 0.00040 |
| Hydrogenophilaceae | 0.00027 | 0.00000 |
| Ilumatobacteraceae | 0.00026 | 0.00000 |
| Lineage_IV_fa | 0.00025 | 0.00000 |
| Candidatus_Zambryskibacteria_fa | 0.00025 | 0.00000 |
| Methyloligellaceae | 0.00024 | 0.00040 |
| Leptospiraceae | 0.00024 | 0.00020 |
| Leptotrichiaceae | 0.00024 | 0.00020 |
| MB-A2-108_fa | 0.00023 | 0.00020 |
| C0119_fa | 0.00023 | 0.00000 |
| Gitt-GS-136_fa | 0.00022 | 0.00079 |
| Anaplasmataceae | 0.00022 | 0.00040 |
| Peptococcaceae | 0.00022 | 0.00020 |
| TRA3-20 | 0.00022 | 0.00000 |
| Chloroflexales_unclassified | 0.00021 | 0.00040 |
| 11-24_fa | 0.00021 | 0.00020 |
| Thermaceae | 0.00021 | 0.00000 |
| Gracilibacteria_unclassified | 0.00021 | 0.00040 |
| Microtrichales_unclassified | 0.00020 | 0.00000 |
| Sneathiellaceae | 0.00020 | 0.00000 |
| Solirubrobacterales_unclassified | 0.00020 | 0.00040 |
| Armatimonadales_fa | 0.00019 | 0.00020 |
| Gracilibacteraceae | 0.00019 | 0.00040 |
| Kiritimatiellaceae | 0.00019 | 0.00020 |
| Hydrogenedensaceae | 0.00019 | 0.00000 |
| Enterococcaceae | 0.00019 | 0.00000 |
| Alcanivoracaceae | 0.00018 | 0.00020 |
| OPB41_fa | 0.00018 | 0.00020 |
| ST-12K33 | 0.00018 | 0.00000 |
| Vermiphilaceae | 0.00017 | 0.00000 |
| DEV007 | 0.00017 | 0.00020 |
| TC1 | 0.00016 | 0.00020 |
| Babeliales_fa | 0.00016 | 0.00020 |
| V2072-189E03_fa | 0.00016 | 0.00040 |
| BRC1_fa | 0.00015 | 0.00000 |
| Planctomycetales_unclassified | 0.00015 | 0.00000 |
| Acidobacteriales_unclassified | 0.00015 | 0.00020 |
| Desulfomicrobiaceae | 0.00015 | 0.00000 |
| Candidatus_Peribacteria_fa | 0.00015 | 0.00000 |
| Rhodothermia_unclassified | 0.00014 | 0.00060 |
| Babeliaceae | 0.00014 | 0.00020 |
| Gallionellaceae | 0.00014 | 0.00020 |
| Gaiellales_fa | 0.00014 | 0.00020 |
| Anaerolineae_unclassified | 0.00014 | 0.00000 |
| Nitrospiraceae | 0.00014 | 0.00020 |
| PeM15_fa | 0.00014 | 0.00000 |
| FTLpost3 | 0.00014 | 0.00020 |
| Thermomicrobiales_unclassified | 0.00013 | 0.00000 |
| Patescibacteria_unclassified | 0.00012 | 0.00000 |
| Erysipelotrichaceae | 0.00012 | 0.00000 |
| UBA12409 | 0.00012 | 0.00020 |
| Listeriaceae | 0.00012 | 0.00020 |
| SM1A07_fa | 0.00011 | 0.00000 |
| DS-100_fa | 0.00011 | 0.00020 |
| Ktedonobacteraceae | 0.00011 | 0.00000 |
| Aminicenantales_fa | 0.00011 | 0.00040 |
| Rarobacteraceae | 0.00011 | 0.00000 |
| Sporolactobacillaceae | 0.00010 | 0.00000 |
| Tistrellaceae | 0.00010 | 0.00000 |
| Bogoriellaceae | 0.00010 | 0.00000 |
| PLTA13_fa | 0.00010 | 0.00000 |
| Armatimonadetes_unclassified | 0.00010 | 0.00020 |
| Chloroflexia_unclassified | 0.00009 | 0.00000 |
| Longimicrobiaceae | 0.00009 | 0.00000 |
| Planctomycetacia_unclassified | 0.00009 | 0.00000 |
| Fodinicurvataceae | 0.00009 | 0.00000 |
| Sulfuricellaceae | 0.00009 | 0.00000 |
| Syntrophaceae | 0.00009 | 0.00000 |
| Leptolyngbyaceae | 0.00009 | 0.00000 |
| Cloacimonadales_unclassified | 0.00009 | 0.00020 |
| Subgroup_5_fa | 0.00009 | 0.00020 |
| Acidothermaceae | 0.00008 | 0.00000 |
| cvE6 | 0.00008 | 0.00000 |
| CCD24_fa | 0.00008 | 0.00000 |
| Cellvibrionales_unclassified | 0.00008 | 0.00000 |
| Victivallales_unclassified | 0.00008 | 0.00020 |
| WS4_fa | 0.00008 | 0.00000 |
| AD3_fa | 0.00007 | 0.00000 |
| Victivallales_fa | 0.00007 | 0.00000 |
| Paenibacillaceae | 0.00007 | 0.00000 |
| Alicyclobacillaceae | 0.00007 | 0.00000 |
| Jonesiaceae | 0.00007 | 0.00020 |
| Clostridia_unclassified | 0.00007 | 0.00000 |
| BRH-c20a_fa | 0.00007 | 0.00000 |
| Coriobacteriia_unclassified | 0.00007 | 0.00000 |
| Marinifilaceae | 0.00007 | 0.00020 |
| Acidobacteria_unclassified | 0.00007 | 0.00020 |
| bac2nit3 | 0.00007 | 0.00000 |
| Armatimonadales_unclassified | 0.00007 | 0.00020 |
| Ignavibacteriaceae | 0.00006 | 0.00020 |
| vadinBE97 | 0.00006 | 0.00020 |
| Chromobacteriaceae | 0.00006 | 0.00000 |
| Porticoccaceae | 0.00006 | 0.00000 |
| Candidatus_Magasanikbacteria_fa | 0.00006 | 0.00000 |
| Candidatus_Moranbacteria_fa | 0.00005 | 0.00000 |
| S085_fa | 0.00005 | 0.00020 |
| Bradymonadales_fa | 0.00005 | 0.00000 |
| Bacilli_unclassified | 0.00005 | 0.00020 |
| Candidatus_Nomurabacteria_fa | 0.00004 | 0.00000 |
| Latescibacteria_fa | 0.00004 | 0.00000 |
| Pla4_lineage_fa | 0.00004 | 0.00020 |
| FCPU426_fa | 0.00004 | 0.00000 |
| Barnesiellaceae | 0.00004 | 0.00000 |
| MWH-CFBk5 | 0.00004 | 0.00000 |
| CPla-3_termite_group | 0.00004 | 0.00000 |
| Omnitrophaceae | 0.00004 | 0.00000 |
| SM1B06 | 0.00004 | 0.00000 |
| Pyrinomonadaceae | 0.00004 | 0.00000 |
| Thermomonosporaceae | 0.00004 | 0.00000 |
| Sporichthyaceae | 0.00004 | 0.00000 |
| NB1-j_fa | 0.00003 | 0.00000 |
| Family_III | 0.00003 | 0.00000 |
| Pseudohongiellaceae | 0.00003 | 0.00000 |
| Segniliparaceae | 0.00003 | 0.00000 |
| LiUU-11-161 | 0.00003 | 0.00000 |
| Muribaculaceae | 0.00003 | 0.00000 |
| Helicobacteraceae | 0.00003 | 0.00000 |
| Azospirillales_Incertae_Sedis | 0.00003 | 0.00000 |
| BSV26 | 0.00002 | 0.00000 |
| SRB2 | 0.00002 | 0.00000 |
| Family_XIII | 0.00002 | 0.00000 |
| Lineage_IIa_fa | 0.00002 | 0.00000 |
| Desulfobacteraceae | 0.00002 | 0.00000 |
| Acidimicrobiia_unclassified | 0.00002 | 0.00000 |
| Candidatus_Kaiserbacteria_fa | 0.00002 | 0.00000 |
| SS1-B-06-26 | 0.00002 | 0.00000 |
| Lentisphaerae_unclassified | 0.00002 | 0.00020 |
| Pasteurellaceae | 0.00002 | 0.00000 |
| Acetobacterales_unclassified | 0.00002 | 0.00000 |
| Cloacimonadaceae | 0.00002 | 0.00000 |
| Criblamydiaceae | 0.00002 | 0.00000 |
| Gemmatimonadetes_unclassified | 0.00002 | 0.00000 |
| Nocardiopsaceae | 0.00001 | 0.00000 |
| Acetobacterales_Incertae_Sedis | 0.00001 | 0.00000 |
| S-70_fa | 0.00001 | 0.00000 |
| Azospirillales_unclassified | 0.00001 | 0.00000 |
| Candidatus_Amesbacteria_fa | 0.00001 | 0.00000 |
| Eggerthellaceae | 0.00001 | 0.00000 |
| Elusimicrobia_unclassified | 0.00001 | 0.00000 |
| Gimesiaceae | 0.00001 | 0.00000 |
| Mollicutes_RF39_fa | 0.00001 | 0.00000 |
| MVP-15_fa | 0.00001 | 0.00000 |
| possible_family_01 | 0.00001 | 0.00000 |


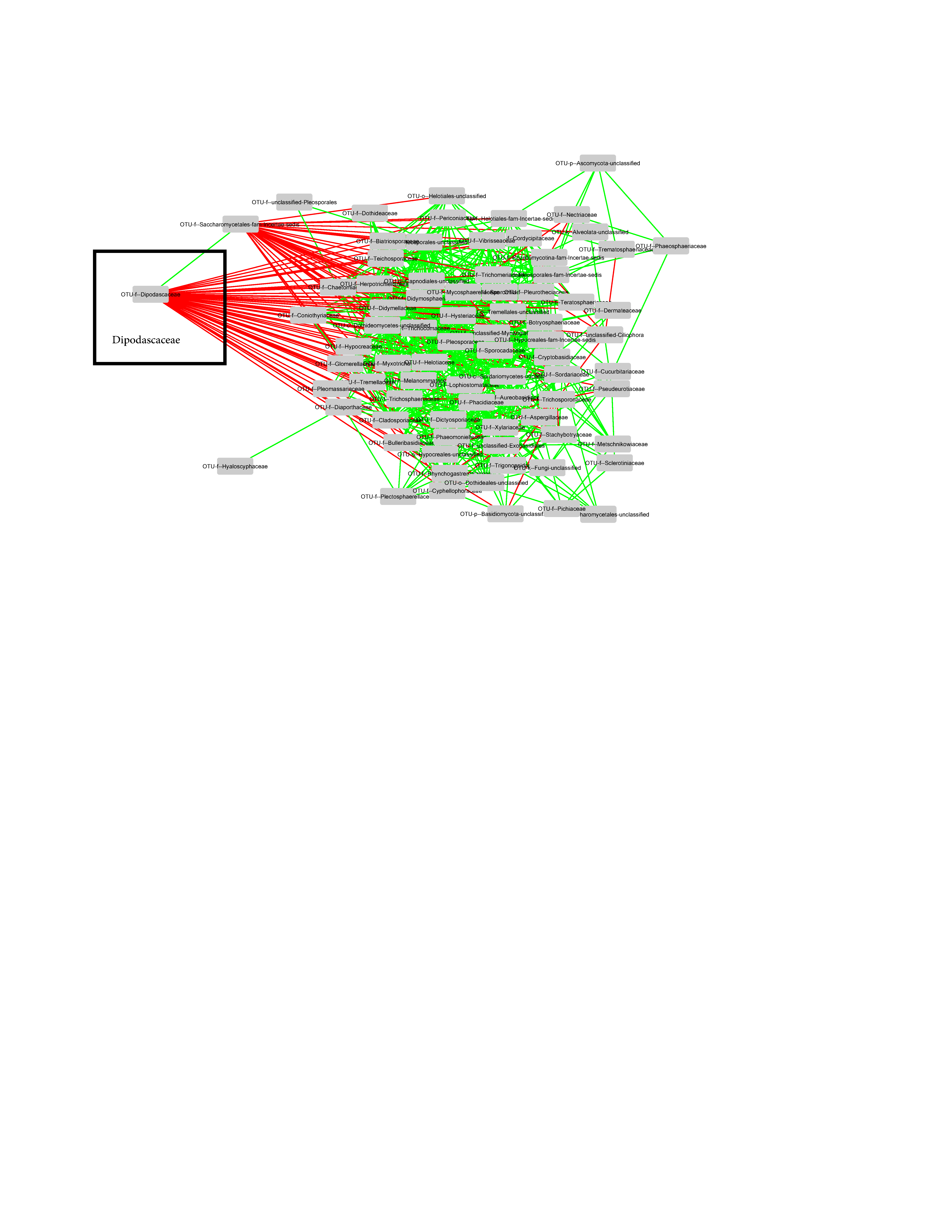


**Figure S1**: Microbial network indicating co-occurrence of fungal families identified in samples collected from all three facilities. Green edges represent positive relationship (co-occurrence) among families, whereas the red edges represent negative relationship (co-exclusion) between two connected nodes.


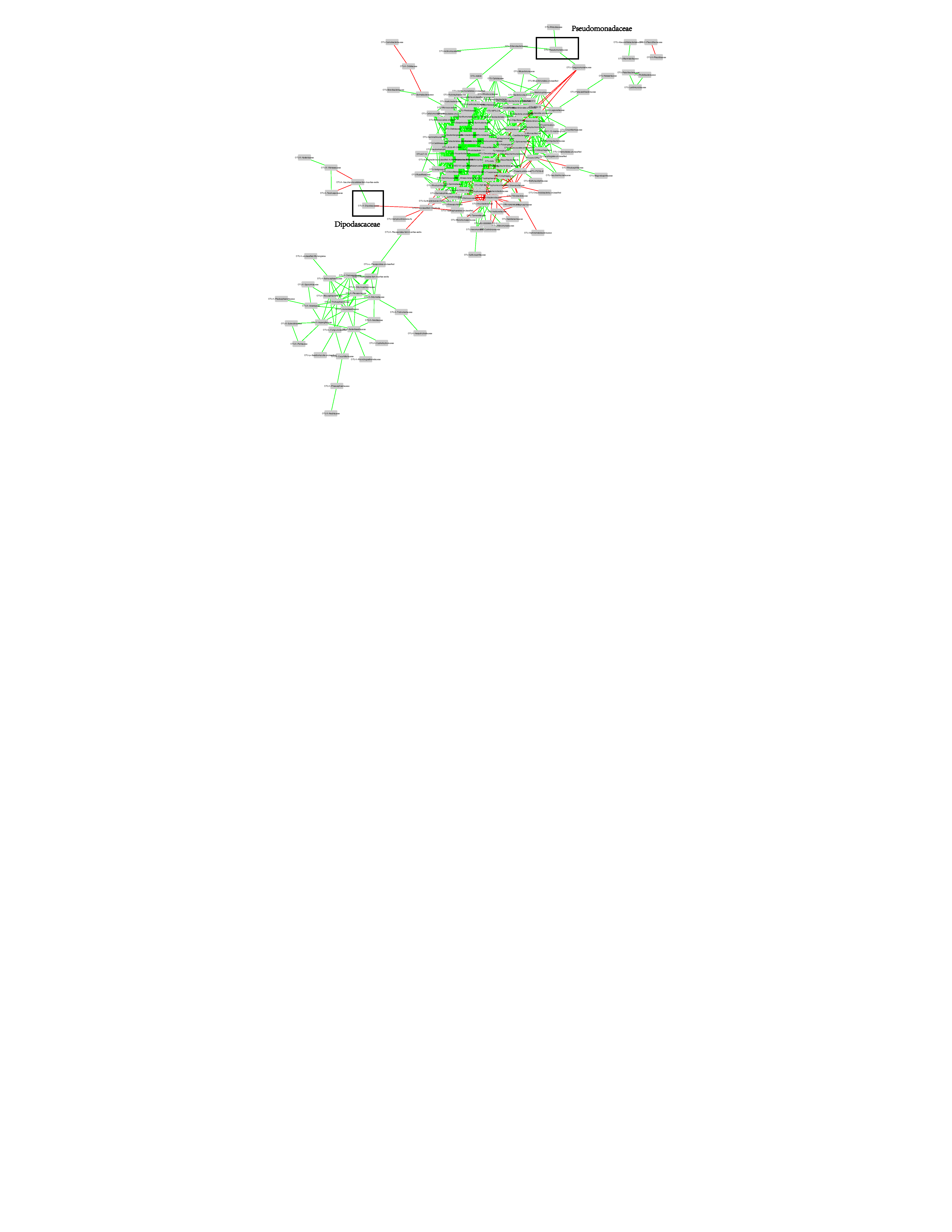


**Figure S2**: Microbial network indicating co-occurrence of bacterial and fungal families in samples collected from three facilities F1, F2, and F3 combined. Green edges represent positive relationship (co-occurrence) among families, whereas the red edges represent negative relationship (co-exclusion) between two connected nodes.

**List L1:** Data analyses workflow

Downloadable workflow file is available on the following link: <https://github.com/kovaclab/Apple-packing-house-environmental-microbiomes/blob/master/Tan%20et%20al.%202019%20data%20anaylsis%20workflow.R>

#Data analysis workflow

#Xiaoqing Tan

#xvt5028@psu.edu

#The Pennsylvania State University

#Microbiome sequence data analyses (carried out in Mothur v 1.39.5)

#From the raw sequencing files (.fastq)

#Set the input directory and number of processing units

#Making.files which include list of samples and the sequences associated with those samples (paired R1, R2)

mothur > make.file(inputdir=., type=fastq, prefix=16s)

#Combine the paired-end reads together

#Extract the sequence and quality score data from fastq files, create the contigs

mothur > make.contigs(file=16s.files)

#Generate descriptive statistics of all sequences in .fasta files

mothur > summary.seqs(fasta=16s.trim.contigs.fasta)

#Filter sequences

##Based on the summary statistics, remove any sequences with ambiguous bases ("N"), shorter than 292, longer than 292

mothur > screen.seqs(fasta=16s.trim.contgis.fasta,

group=16s.contigs.groups, summary=16s.trim.contigs.summary,

minlength=292, maxlenth=292, maxambig=0)

#Remove identical (grouped) sequences; representative sequence will be picked and stored in .fasta and corresponding sequences will be saved as sequence names to reduce computational work

mothur > unique.seqs(fasta=16s.trim.contigs.good.fasta)

#Create a count table of current unique sequences

mothur > count.seqs(name=16s.trim.contigs.good.names,

group=16s.contigs.good.groups)

#Analyze the target 16S rRNA V4 region

#Customize database to target V4 region

#Define start and end positions within a 16S rRNA sequence

#Set keepdots=F to false to remove output trailing dots from fragments

mothur > pcr.seqs(fasta=silva.nr_v132.align, start=11894, end=25319,

keepdots=F, processors=8)

#Rename reference files

mothur > rename.file(input = silva.bacteria.pcr.fasta, new=

silva.v4.fasta)

#Align the target region to SILVA reference database

#Determined k size (ksize=); default is 8

#Allow reverse matching to the reference using flip=T

mothur > align.seqs(fasta=16s.trim.contigs.good.unique.fasta,

reference=silva.v4.fasta, flip=T)

#Generate descriptive statistics for all sequences in .fasta files using summary.seqs

mothur > summary.seqs(fasta=16s.trim.contigs.good.unique.align,

count=16s.trim.contigs.good.count_table)

#Remove sequences that are before or after the sites of alignment from the previous step

mothur > screen.seqs(fasta=16s.trim.contgis.good.unique.align,

group=16s.contigs.good.count_table,

summary=16s.trim.contigs.good.unique.summary, minlength=Undecided,

maxlenth=Undecided, maxhomop=8)

#Remove overhangs

#Remove the alignment characters that only consist of "-", using vertical=T

#Remove the sequences containing '.' by using trump=.

mothur > filter.seqs(fasta=16s.trim.contigs.good.unique.good.align,

vertical=T, trump=.)

#Rerun unique.seqs in case new redundant sequences were created by filtering

mothur > unique.seqs(fasta=16s.trim.contigs.good.unique.good.filter.fasta,

count = 16s.trim.contigs.good.good.count_table)

#De-noise sequences

#Set diffs=2 as a threshold of mismatches in the sequence

mothur > pre.cluster(fasta=16s.trim.contigs.good.unique.good.filter.unique.fast

a, count=16s.trim.contigs.good.unique.good.filter.count_table,

diffs=2)

#Read .fasta and count file to chimera sequences

mothur > chimera.vsearch(fasta=16s.trim.contigs.good.unique.good.filter.unique.p

recluster.fasta,

count=16s.trim.contigs.good.unique.good.filter.unique.precluster.count

_table, dereplicate=t)

#Remove chimera

mothur > remove.seqs(fasta=16s.trim.contigs.good.unique.good.filter.unique.prec

luster.fasta,

accnos=16s.trim.contigs.good.unique.good.filter.unique.precluster.deno

vo.vsearch.accnos)

#Assign taxonomy

mothur > classify.seqs(fasta=16s.trim.contigs.good.unique.good.filter.unique.pr

ecluster.pick.fasta,

count=16s.trim.contigs.good.unique.good.filter.unique.precluster.denov

o.uchime.pick.count_table, reference=silva.nr_v123.align,

taxonomy=silva.nr_v123.tax)

#Remove chloroplast and mitochondria sequences

mothur > remove.lineage(fasta=16s.trim.contigs.good.unique.good.filter.unique.p

recluster.pick.fasta,

count=16s.trim.contigs.good.unique.good.filter.unique.precluster.denov

o.uchime.pick.count_table,

taxonomy=16s.trim.contigs.good.unique.good.filter.unique.precluster.pi

ck.nr_v132.wang.taxonomy, taxon=Chloroplast-Mitochondria-unknown-

Eukaryota)

#Calculate uncorrected pairwise distances between aligned DNA sequences. By default, a gap is penalized; cutoff value indicates that distances larger than 0.03 (>97% similarity) will not be saved

mothur > dist.seqs(fasta=16s.trim.contigs.good.unique.good.filter.unique.preclu

ster.pick.pick.fasta, cutoff=0.03)

#Assign sequences to OTUs, Mothur provides three different methods of alignment. By default, opticlust method is used.

mothur > cluster(column=16s.trim.contigs.good.unique.good.filter.unique.preclus

ter.pick.pick.dist, count =

16s.trim.contigs.good.unique.good.filter.unique.precluster.denovo.vsea

rch.pick.pick.count_table)

#Determine how many sequences are assigned to each OTU at the 0.03 cutoff. Distribute OTUs into groups

#The output shared file is used as an OTU table

mothur > make.shared(list=16s.trim.contigs.good.unique.good.filter.unique.precl

uster.pick.pick.opti_mcc.list,

count=16s.trim.contigs.good.unique.good.filter.unique.precluster.denov

o.vsearch.pick.pick.count_table, label=0.03)

#Determine taxonomy for all OTUs. Output of this command is a taxonomy file

mothur > classify.otu(list=16s.trim.contigs.good.unique.good.filter.unique.prec

luster.pick.pick.opti_mcc.list,

count=16s.trim.contigs.good.unique.good.filter.unique.precluster.denov

o.vsearch.pick.pick.count_table,

taxonomy=16s.trim.contigs.good.unique.good.filter.unique.precluster.pi

ck.pds.wang.pick.taxonomy, label=0.03)

####Downstream analysis###

#Plot L. monocytogenes occurrence in three facilities using a bar plot

#Load the file containing data about facility and L. monocytogenes occurrence

lm <- read.csv(file.choose(), header = T)

lmoccurrence <- ggplot(data = lm, aes(x=Facility, y=Number.of.samples,fill=L..monocytogenes))+

geom_bar(stat = "identity",width = 0.5)+

geom_text(aes(label=Number.of.samples, size=3), hjust=0.5, vjust=3) +

theme_bw(base_size = 12)+

theme(legend.text=element_text(size=15), legend.title= element_text(size=15)) +

theme(axis.text.x = element_text(size=13), axis.text.y = element_text(size=13)) +

theme(axis.title = element_text(size=15)) +

scale_x_discrete(name = "Facility") +

scale_y_discrete(name = "Number of Samples")

ggsave("lmoccurrence.pdf", plot = lmoccurrence, device="pdf", width=10, height=7, units="in",dpi=600)

#Plot the PCoA

#Obtain required R packages

library(phyloseq)

library(ape)

library(vegan)

library(ggplot2)

#Import data

set.seed(336)

otus <- import_mothur(mothur_shared_file= file.choose())

otus2 <- as.data.frame(otus)

otus.t <- t(otus)

min(rowSums(otus.t))

otus.r <- rrarefy(otus.t,4501)

OTU <- otu_table(otus.r , taxa_are_rows=FALSE)

taxon <- import_mothur(mothur_constaxonomy_file = file.choose())

taxon <- as.data.frame(taxon)

colnames(taxon) <- c("Domain", "Phylum", "Class", "Order", "Family", "Genus")

TAX = tax_table(as.matrix(taxon))

metadat <- read.table(file.choose(), sep=",", header=T, row.names=1)

META = sample_data(metadat)

#Plot PCoA for rarefied samples (16S rRNA data)

phyloseq = phyloseq(OTU, TAX, META)

TREE = rtree(ntaxa(phyloseq), rooted=TRUE, tip.label = taxa_names(phyloseq))

phyloseq = phyloseq(OTU,TAX,META,TREE)

phyloseq

ord = ordinate (phyloseq, "PCoA", "unifrac", weighted = TRUE)

po = plot_ordination(phyloseq, ord, color="Facility",shape="L..monocytogenes")

PCOA16S <- po +

geom_point(size=4)+theme_classic() +

theme(legend.text=element_text(size=13), legend.title= element_text(size=15)) +

theme(axis.text.x = element_text(size=13), axis.text.y = element_text(size=13)) +

theme(axis.title = element_text(size=15)) +

scale_x_continuous(name = "PC1 (10.7%)") +

scale_y_continuous(name = "PC2 (6.6%)")

PCOA16S

#Import data for ITS

set.seed(336)

otus_ITS <- import_mothur(mothur_shared_file= file.choose())

otus2_ITS <- as.data.frame(otus_ITS)

otus.t_ITS <- t(otus_ITS)

min(rowSums(otus.t_ITS))

otus.r_ITS <- rrarefy(otus.t_ITS,5323)

OTU_ITS <- otu_table(otus.r_ITS , taxa_are_rows=FALSE)

taxon_ITS <- import_mothur(mothur_constaxonomy_file = file.choose())

taxon_ITS <- as.data.frame(taxon_ITS)

colnames(taxon_ITS) <- c("Domain", "Phylum", "Class", "Order", "Family", "Genus")

TAX_ITS = tax_table(as.matrix(taxon_ITS))

metadat_ITS <- read.table(file.choose(), sep=",", header=T, row.names=1)

META_ITS = sample_data(metadat_ITS)

#Plot PCoA for rarefied samples (ITS data)

phyloseq_ITS = phyloseq(OTU, TAX, META)

TREE_ITS = rtree(ntaxa(phyloseq_ITS), rooted=TRUE, tip.label = taxa_names(phyloseq_ITS))

phyloseq_ITS = phyloseq(OTU,TAX,META,TREE_ITS)

phyloseq_ITS

ord_ITS = ordinate (phyloseq_ITS, "PCoA", "unifrac", weighted = TRUE)

po_ITS = plot_ordination(phyloseq_ITS, ord_ITS, color="Facility",shape="L.monocytogenes")

PCOAITS <- po_ITS +

geom_point(size=4)+theme_classic() +

theme(legend.text=element_text(size=13), legend.title= element_text(size=15)) +

theme(axis.text.x = element_text(size=13), axis.text.y = element_text(size=13)) +

theme(axis.title = element_text(size=15)) +

scale_x_continuous(name = "PC1 (43.1%)") +

scale_y_continuous(name = "PC2 (20.5%)")

PCOA combined plot

#Combine the 16S rRNA and ITS PCoA plots

a = plot_grid(PCOA16S + theme(legend.position= "none") , PCOAITS + theme(legend.position = "none") ,

ncol=1, nrow=2, labels=c("A", "B"), label_size = 20)

b = get_legend(PCOAITS)

c = plot_grid(a, b, ncol=2, rel_widths = c(3,1))

c

ggsave("PCOAcombined.pdf", plot =c, device="pdf", width=10, height=10, units="in",dpi=600)

ggsave("PCOAcombined.png", plot =c, device="png", width=10, height=10, units="in",dpi=600)

#Stack barplot for bacterial and fungal communities at a family level

#Use phyoseq objects for 16S rRNA and ITS

#Melt to long format (for ggploting)

family_16 <- phyloseq %>%

tax_glom(taxrank = "Family") %>% #agglomerate at a family level

transform_sample_counts(function(x) {x/sum(x)} ) %>% #Transform to relative abundance

psmelt() %>% #Melt to long format

arrange(Family) #Sort data frame alphabetically by phylum

family_ITS <- phyloseq_ITS %>%

tax_glom(taxrank = "Rank5") %>%

transform_sample_counts(function(x) {x/sum(x)} ) %>%

psmelt() %>%

arrange(Family)

write.csv(family_16, "combined_family_16.csv")

write.csv(family_ITS, "combined_family_ITS.csv") #Write the filtered file into csv format

#Filter the 'Abundance' column to 'less than' 0.10 (in Excel)

#All of the outcome rows are representative families with abundance lower than 0.10

#Change all column labels under 'Family' to "Other"

#Save as .csv file

#Open file in R

#Figure 1: Facility vs. L. monocytogenes

combined_family_16s <- read.csv(file.choose(), sep=",", header=T, row.names=1)

Family_colors <- c("#FFF5F0","#525252","#CB181D","#99000D","#EF3B2C","#FB6A4A","#FC9272","#FEE0D2","#4292C6","#084594","#FFF5F0","#EF3B2C","#9ECAE1","#C6DBEF","#DEEBF7","#6BAED6","#2171B5","#737373")

#Plot Figure 1

fig1 <- ggplot(combined_family_16s, aes(x = SampleOrder, y = Abundance , fill = Family)) +

facet_grid(Facility~Lmono)+

geom_bar(stat = "identity") +

scale_fill_manual(values = Family_colors) +

theme(legend.text=element_text(size=13), legend.title= element_text(size=15)) +

theme(axis.title.x = element_blank(), axis.text.x=element_blank(), axis.ticks.x=element_blank(),

axis.title.y=element_text(size=15)) +

guides(fill = guide_legend(reverse = FALSE, keywidth = 1, keyheight = 1, ncol=1)) +

ylab("Relative Abundance") + theme(panel.background = element_rect(fill="transparent", color =NA),

plot.background = element_rect(fill="transparent", color =NA)) +

theme(strip.background= element_blank(), strip.text = element_text(size=15),

panel.border = element_rect(color="black", fill=NA))

fig1

ggsave("figure1.pdf", plot =fig1, device="pdf", width=8, height=5, units="in",dpi=600)

#Figure 2: Facility vs. section

combined_family_16s <- read.csv(file.choose(), sep=",", header=T, row.names=1)

Family_colors <- c("#FFF5F0","#525252","#CB181D","#99000D","#EF3B2C","#FB6A4A","#FC9272","#FEE0D2","#4292C6","#084594","#FFF5F0","#EF3B2C","#9ECAE1","#C6DBEF","#DEEBF7","#6BAED6","#2171B5","#737373")

#Plot Figure 2

fig2 <- ggplot(combined_family_16s, aes(x = SampleOrder, y = Abundance , fill = Family)) +

facet_grid(Facility~Section)+

geom_bar(stat = "identity") +

scale_fill_manual(values = Family_colors) +

theme(legend.text=element_text(size=13), legend.title= element_text(size=15)) +

theme(axis.title.x = element_blank(), axis.text.x=element_blank(), axis.ticks.x=element_blank(),

axis.title.y=element_text(size=15)) +

guides(fill = guide_legend(reverse = FALSE, keywidth = 1, keyheight = 1, ncol=1)) +

ylab("Relative Abundance") + theme(panel.background = element_rect(fill="transparent", color =NA),

plot.background = element_rect(fill="transparent", color =NA)) +

theme(strip.background= element_blank(), strip.text = element_text(size=15),

panel.border = element_rect(color="black",fill=NA))

fig2

ggsave("figure2.pdf", plot =fig1, device="pdf", width=8, height=5, units="in",dpi=600)

ggsave("figure2.png", plot =fig1, device="png", width=8, height=5, units="in",dpi=600)

library(cowplot)

a = plot_grid(fig1 + theme(legend.position= "none") , fig2 + theme(legend.position = "none") ,

ncol=1, nrow=2, labels=c("A", "B"), label_size = 20)

b = get_legend(fig2)

c = plot_grid(a, b, ncol=3, rel_widths = c(10,1))

c

ggsave("figre_combine.pdf", plot=c, device="pdf", width=10, height=7, units="in", dpi=600)

ggsave("figre_combine.png", plot=c, device="png", width=10, height=7, units="in", dpi=600)

#Plot a stack bar plot based on ITS data

combined_family_ITS <- read.csv(file.choose(), sep=",", header=T, row.names=1)

Family_colors <- c("#084594", "#2171B5", "#4292C6","#9ECAE1","#FFF5F0","#C6DBEF", "#DEEBF7","#6BAED6" ,"#99000D","#EF3B2C","#FC9272","#F7FBFF","#FB6A4A", "#FCBBA1", "#FEE0D2","#525252","#737373","#CB181D","#FFDAB9","#E6E6FA")

#Plot facility vs. L. monocytogenes occurence

ITSfacilitystack <- ggplot(combined_family_ITS, aes(x = SampleOrder, y = Abundance, fill = Family)) + facet_grid(Facility~L.monocytogenes)+

geom_bar(stat = "identity") +

geom_bar(stat = "identity") +

scale_fill_manual(values = Family_colors) +

theme(legend.text=element_text(size=13), legend.title= element_text(size=15)) +

theme(axis.title.x = element_blank(), axis.text.x=element_blank(), axis.ticks.x=element_blank(),

axis.title.y=element_text(size=15)) +

guides(fill = guide_legend(reverse = FALSE, keywidth = 1, keyheight = 1, ncol=1)) +

ylab("Relative Abundance") + theme(panel.background = element_rect(fill="transparent", color =NA),

plot.background = element_rect(fill="transparent", color =NA)) +

theme(strip.background= element_blank(), strip.text = element_text(size=15),

panel.border = element_rect(color="black", fill=NA))

#Plot Facility vs. Sections

ITSsectionstack <- ggplot(combined_family_ITS, aes(x = SampleOrder, y = Abundance, fill = Family)) + facet_grid(Facility~Section) +

geom_bar(stat = "identity") +

scale_fill_manual(values = Family_colors) +

theme(legend.text=element_text(size=13), legend.title= element_text(size=15)) +

theme(axis.title.x = element_blank(), axis.text.x=element_blank(), axis.ticks.x=element_blank(),

axis.title.y=element_text(size=15)) +

guides(fill = guide_legend(reverse = FALSE, keywidth = 1, keyheight = 1, ncol=1)) +

ylab("Relative Abundance") + theme(panel.background = element_rect(fill="transparent", color =NA),

plot.background = element_rect(fill="transparent", color =NA)) +

theme(strip.background= element_blank(), strip.text = element_text(size=15),

panel.border = element_rect(color="black", fill=NA))

a_ITS = plot_grid(ITSfacilitystack + theme(legend.position= "none") , ITSsectionstack + theme(legend.position = "none") ,

ncol=1, nrow=2, labels=c("A", "B"), label_size = 20)

b_ITS = get_legend(ITSfacilitystack)

c_ITS = plot_grid(a_ITS, b_ITS, ncol=3, rel_widths = c(5,1))

c_ITS

#Save and export the figure

ggsave("ITSstackcombined.pdf", plot=c, device="pdf", width=12, height=10, units="in", dpi=600)

#Making phyloseq object for rarefaction curve before normalization

phyloseq_rare_16s = phyloseq(otu_table(otus.t, taxa_are_rows=FALSE), TAX, META)

phyloseq_rare_ITS = phyloseq(otu_table(otus.t_ITS, taxa_are_rows=FALSE), TAX_ITS, META_ITS)

#Rarefaction curves

rare_16s_apple_plot <- ggrare(phyloseq_rare_16s, step = 100, se= TRUE, color="Facility")

rare_16s_byfacility_plot <- rare_16s_apple_plot + facet_grid(Facility~.) +

theme(strip.text.y=element_blank()) +xlab("Number of OTUs") + ylab("Number of unique OTUs") +

scale_x_continuous(breaks= seq(0,180000, 10000)) + theme(axis.text.x = element_text(size=10, angle=90)) +

annotate("segment", x=-Inf, xend=Inf, y=-Inf, yend= -Inf) +

annotate("segment", x=-Inf, xend=Inf, y=-Inf, yend= -Inf) +

annotate("segment", x=-Inf, xend=Inf, y=-Inf, yend= -Inf)

rare_ITS_apple_plot <- ggrare(phyloseq_rare_ITS, step = 100, se= TRUE, color="Facility")

rare_ITS_byfacility_plot <- rare_ITS_applot_plot + facet_grid(Facility ~ .) +

theme(strip.text.y=element_blank()) +xlab("Number of OTUs") + ylab("Number of unique OTUs") +

scale_x_continuous(breaks= seq(0,400000, 20000)) + theme(axis.text.x = element_text(size=10, angle=90)) +

annotate("segment", x=-Inf, xend=Inf, y=-Inf, yend= -Inf) +

annotate("segment", x=-Inf, xend=Inf, y=-Inf, yend= -Inf) +

annotate("segment", x=-Inf, xend=Inf, y=-Inf, yend= -Inf)

rarefig <- plot_grid(rare_16s_byfacility_plot, rare_ITS_byfacility_plot, nrow=1, ncol=2, labels=c("A", "B"), label_size = 20)

ggsave("rarefig.pdf", plot=rarefig, device="pdf", width=11, height=6, units="in", dpi=600)

ggsave("rarefig.png", plot=rarefig, device="png", width=11, height=6, units="in", dpi=600)

#Alpha diversity

alpha <-estimate_richness(phyloseq, measures=c("Shannon", "InvSimpson", "Chao1"))

estimate_richness(phyloseq, split= TRUE, measures=c("Chao1", "Shannon", "InvSimpson"))

#Import 16S rRNA data

alpha_16s <- read.csv(file.choose(), sep = ",", header = T, row.names = 1)

alpha_ITS <- read.csv(file.choose(), sep = ",", header = T, row.names = 1)

#Pairwise.t.test for alpha diversity using Shannon and Inverse Simpson indices

pairwise.t.test(alpha_16s$Shannon, alpha_16s$Facility, p.adjust.method = "bonferroni")

pairwise.t.test(alpha_16s$InvSimpson, alpha_16s$Facility, p.adjust.method = "bonferroni")

#Import ITS data

alpha_ITS <- read.csv(file.choose(), sep = ",", header = T, row.names = 1)

#Pairwise.t.test for alpha diversity using Shannon and Inverse Simpson indices

pairwise.t.test(alpha_ITS$Shannon, alpha_ITS$Facility, p.adjust.method = "bonferroni")

pairwise.t.test(alpha_ITS$InvSimpson, alpha_ITS$Facility, p.adjust.method = "bonferroni")

#Violin plots for alpha diversity

library(ggpubr)

#16S rRNA alpha diversity violin plots

alpha_16s <- read.csv(file.choose(), sep = ",", header = T, row.names = 1)

alpha16s1 <- ggviolin(alpha_16s, x = "Facility", y = "Shannon", add = "boxplot",

fill= "Facility" ) +

theme(axis.text.x = element_text(size=13), axis.text.y = element_text(size=13)) +

theme(axis.title = element_text(size=15))

alpha16s2 <- ggviolin(alpha_16s, x = "Facility", y = "InvSimpson", add = "boxplot",

fill = "Facility" ) +

theme(axis.text.x = element_text(size=13), axis.text.y = element_text(size=13)) +

theme(axis.title = element_text(size=15))

#ITS alpha diversity violin plots

alpha_ITS <- read.csv(file.choose(), sep = ",", header = T, row.names = 1)

alphaITS1 <- ggviolin(alpha_ITS, x = "Facility", y = "Shannon", add = "boxplot",

fill= "Facility" ) +

theme(axis.text.x = element_text(size=13), axis.text.y = element_text(size=13)) +

theme(axis.title = element_text(size=15))

alphaITS2 <- ggviolin(alpha_ITS, x = "Facility", y = "InvSimpson", add = "boxplot",

fill = "Facility" ) +

theme(axis.text.x = element_text(size=13), axis.text.y = element_text(size=13)) +

theme(axis.title = element_text(size=15))

#Combined 16S and ITS alpha diversity plots

a = plot_grid(alpha16s1 + theme(legend.position= "none") ,

alpha16s2 + theme(legend.position = "none") ,

alphaITS1 + theme(legend.position = "none"),

alphaITS2 + theme(legend.position = "none"),

ncol=2, nrow=2, labels=c("A", "B","C","D"), label_size = 20)

ggsave("alphadiversity.pdf", plot =a, device="pdf", width=12, height=10, units="in",dpi=600)

ggsave("alphadiversity.png", plot =a, device="png", width=12, height=10, units="in",dpi=600)

#Pairwise PERMANOVA

library(devtools)

install_github("pmartinezarbizu/pairwiseAdonis/pairwiseAdonis")

library(pairwiseAdonis)

#Run pairwise PERMANOVA for 16S rRNA data

permanova_data_16s <- data.frame(sample_data(phyloseq))

pairwise_perm_16s_f <- pairwise.adonis(otu_table(phyloseq), permanova_data_16s$Facility)

pairwise_perm_16s_s <- pairwise.adonis(otu_table(phyloseq), permanova_data_16s$Section)

#Export the file in .csv

write.csv(pairwise_perm_16s_f, "microbiome_pairwise_Facility.csv")

write.csv(pairwise_perm_16s_s, "microbiome_pairwise_Section.csv")

#Run pairwise PERMANOVA based on ITS data

permanova_data_ITS <- data.frame(sample_data(phyloseq))

pairwise_perm_ITS_f <- pairwise.adonis(otu_table(phyloseq), permanova_data_ITS$Facility)

pairwise_perm_ITS_s <- pairwise.adonis(otu_table(phyloseq), permanova_data_ITS$Section)

#Export the file in .csv

write.csv(pairwise_perm_ITS_f, "mycobiome_pairwise_Facility.csv")

write.csv(pairwise_perm_ITS_s, "mycobiome_pairwise_Section.csv")

#PICRUSt analysis plot

#Import csv file for picrust, actural abundance

picrustfuntion <- read.csv(file.choose(), sep = ",", header = T)

#Make boxplots based on PICRUSt data

allfunctionabun <- ggplot(picrustfuntion, aes(x=Facility, y=Abundance, fill=Pathway.category)) +

theme_bw() +geom_boxplot()+

theme(legend.text=element_text(size=13), legend.title= element_text(size=15)) +

theme(axis.title.x = element_text(size=15),

axis.title.y=element_text(size=15))

#Make a plot based on PICRUSt function abundance

functionabundance <- ggplot(picrustfuntion, aes(x=Facility, y=Abundance, fill=Pathway.category)) +theme_bw() +geom_boxplot()

#Import .csv file for PICRUSt, relative abundance, all combined

picrustrelabun <- read.csv(file.choose(), sep = ",", header = T)

refunctionabun <- ggplot(picrustrelabun, aes(x=Facility, y=relative.abundance, fill=Category)) +theme_bw() +

geom_boxplot() +

theme(legend.text=element_text(size=13), legend.title= element_text(size=15)) +

theme(axis.title.x = element_text(size=15),

axis.title.y=element_text(size=15))

#Create a plot for functional categories based on relative abundance

re_all_plot <-ggplot(picrustrelabun, aes(x=Facility, y=relative.abundance, fill=Category)) +theme_bw() + geom_boxplot()

#Import csv file for PICRUSt, relative abundance, by category

picrustfuntioncate <- read.csv(file.choose(), sep = ",", header = T)

#Pairwise.t.test for significant difference between categories

pairwise.t.test(picrustfuntioncate$Metabolism, picrustfuntioncate$Facility, p.adjust.method = "bonferroni")

ggplot(picrustfuntioncate, aes(x=Facility, y=Cellular.Processes)) + theme_bw() + geom_col()

#Plot metabolism and environment functional categories for each facility

metabolism <- ggplot(picrustfuntioncate, aes(x=Facility, y=Metabolism)) + theme_bw() +

geom_boxplot() +

theme(legend.text=element_text(size=13), legend.title= element_text(size=15)) +

theme(axis.title.x = element_text(size=15),

axis.title.y=element_text(size=15))

Environment <- ggplot(picrustfuntioncate, aes(x=Facility, y=Environmental.Information.Processing)) +

theme_bw() + geom_boxplot() +

theme(legend.text=element_text(size=13), legend.title= element_text(size=15)) +

theme(axis.title.x = element_text(size=15),

axis.title.y=element_text(size=15))

a = plot_grid(allfunctionabun, refunctionabun, nrow=2, labels=c("A", "B"), label_size = 20)

b =plot_grid(metabolism, Environment,nrow = 2, labels = c("C","D"), label_size = 20)

c = plot_grid(a, b, ncol=2, rel_widths = c(6,3))

c

ggsave("picrust.pdf", plot=c, device="pdf", width=12, height=10, units="in", dpi=600)

ggsave("picrust.png", plot=c, device="png", width=12, height=10, units="in", dpi=600)
